# Multifaceted Biological Activities of Culinary Herb and Spice Extracts: In Vitro and In Silico Simulation Insights into Inflammation-Related Targets

**DOI:** 10.3390/foods14091456

**Published:** 2025-04-23

**Authors:** Nance Hontman, Jéssica Gonçalves, José S. Câmara, Rosa Perestrelo

**Affiliations:** 1 CQM—Centro de Química da Madeira, Universidade da Madeira, Campus da Penteada, 9020-105 Funchal, Portugal; 2 Departamento de Química, Faculdade de Ciências Exatas e Engenharia, Universidade da Madeira, Campus da Penteada, 9020-105 Funchal, Portugal

**Keywords:** natural extracts, volatilomic fingerprint, HS-SPME/GC-MS, antioxidant, anti-inflammatory

## Abstract

Culinary herbs and spices are valued worldwide for their flavor, aroma, and medicinal benefits. They encompass diverse bioactive metabolites, such as polyphenols and terpenoids, which contribute to plant defense and offer anticarcinogenic, anti-inflammatory, antioxidant, and cognitive-enhancing effects. This study aimed to establish the volatile fingerprint of culinary herbs (lemon verbena, chives, basil, sage, coriander, and parsley) and spices (curcuma, nutmeg, cumin, black pepper, Jamaica pepper, and juniper berry) using headspace solid-phase microextraction combined with gas chromatography-mass spectrometry (HS-SPME/GC-MS). The predominant volatile organic metabolites (VOMs) identified were subjected to in silico molecular docking simulations of anti-Alzheimer’s (e.g., acetylcholinesterase (AChE), butyrylcholinesterase (BChE)), antioxidants (e.g., monoamine oxidase B (MAO-B), inducible nitric oxide synthase (iNOS)), and anti-inflammatory receptors (e.g., 5-lipoxygenase (5-LOX), cyclooxygenase-2 (COX-2)). The culinary herb and spice extracts were also subjected to in vitro assays to evaluate their potential as antioxidant (DPPH, ABTS, and ORAC) and anti-inflammatory (% protein denaturation) agents. A total of 121 VOMs were identified in the culinary herbs and spices, with the predominant chemical families being monoterpenoids (48.3%), sesquiterpenoids (14.0%), esters (11.9%), and carbonyl compounds (8.8%). In silico molecular docking simulations revealed that cuminaldehyde, β-caryophyllene, γ-curcumene, germacrene D, and τ-cadinol exhibited the strongest inhibitory activities against the selected receptors. Among the extracts, Jamaica pepper showed the highest antioxidant and anti-inflammatory activities, while lemon verbena exhibited the lowest ones. These findings highlight the promising potential of the studied culinary herbs and spices in the modulation of inflammatory processes related to Alzheimer’s disease. However, further investigations, particularly clinical studies, are recommended to validate these results and explore their therapeutic applications.

## 1. Introduction

A growing body of research supports the use of culinary herbs and spices as preventive and therapeutic agents in medicine, emphasizing their numerous health benefits. These benefits are primarily attributed to the abundance of phytochemicals they contain, particularly secondary bioactive metabolites. These metabolites, such as volatile organic compounds (VOMs), polyphenols, anthocyanins, tannins, alkaloids, and vitamins, have demonstrated the ability to prevent and alleviate both acute and chronic diseases due to their antioxidant, anti-inflammatory, and antimicrobial properties [1,2,3,4]. Thus, the consumption of culinary herbs and spices has been linked to mood and cognition enhancement, antitumorigenic and anticarcinogenic effects, as well as glucose- and cholesterol-lowering properties. The literature has reported the bioactivities of VOMs identified in culinary herbs and spices considered in the current study, along with their known biological effects. For instance, citral and limonene from lemon verbena (*Aloysia citrodora*) have shown antioxidant and antimicrobial properties; eugenol and linalool from basil (*Ocimum basilicum* L.) are noted for their anti-inflammatory effects; and α-pinene and geranyl acetate from coriander (*Coriandrum sativum* L.) exhibit analgesic and antimicrobial action. Similar bioactive profiles are seen across other spices such as curcuma (*Curcuma longa* L.), nutmeg (*Myristica fragrans*), cumin (*Cuminum cyminum*), black pepper (*Piper nigrum* L.), Jamaica pepper (*Pimenta dioica* L.), and juniper berry (*Juniperus communis* L.) [5,6,7,8,9,10,11,12,13]. Several extraction procedures have been proposed to extract VOMs from culinary herbs and spices, such as hydrodistillation [14], microwave-assisted hydrodistillation [13,14], Soxhlet [15], maceration [15], and supercritical fluid extraction [16]. Advancements in technology have shown weaknesses in prior extraction procedures, such as low stability and being time-consuming and solvent-intensive, among others [13]. Therefore, optimizing a useful and effective instrument for extracting and detecting VOMs in culinary herbs and spices is critical. HS-SPME has gained popularity for extracting VOMs because of its ease of use, lack of solvents, high sensitivity, and reproducibility [7,8,17]. Wei et al. [13] found that integrating sampling, isolation, concentration, and injection into a single phase significantly reduced the time required to determine VOM fingerprints in culinary herbs and spices. Moreover, HS-SPME can be combined with gas chromatography coupled with mass spectrometry (GC-MS), which is an analytical approach that integrates the characteristics of gas chromatography with the sensitivity and selective capacity of the mass detector, allowing for the identification and quantification of VOMs in complex mixtures. This effective analytical approach improves the recovery of VOMs through adsorption on fused silica fibers, with minimal sample preparation [18]. Several studies have demonstrated the utility of HS-SPME/GC-MS for profiling VOMs in herbs such as lemon verbena, basil, coriander, and black pepper [7,8,13,17,18,19]. In addition, the key VOMs identified in culinary herbs and spices can be subjected to in silico molecular docking simulations to predict their interactions with Alzheimer’s-related targets (e.g., acetylcholinesterase (AChE) and β-secretase) and oxidative/inflammatory pathways (e.g., inducible nitric oxide synthase (iNOS), 5-lipoxygenase (5-LOX), and cyclooxygenase-2 (COX-2)). This approach, combined with in vitro assays (e.g., antioxidant and anti-inflammatory assays), could streamline the discovery of bioactive compounds for neurodegenerative and related diseases by prioritizing candidates with the highest predicted efficacy for further experimental testing. Mostafa et al. [20] evaluated the neuroprotective effects of black pepper cold-pressed oil (CPO) against scopolamine-induced oxidative stress and memory impairment in rats using a combination of GC-MS and in silico molecular docking studies. Their findings revealed that CPO administration reduced AChE levels in the hippocampi of scopolamine-treated rats by 51%, which was corroborated by molecular docking studies that highlighted the potential of key VOMs in CPO to interact with Alzheimer’s-related targets and counteract oxidative stress.

Based on the points discussed above, the current research aims to provide a multidisciplinary approach that integrates volatile fingerprints of culinary herbs and spices using HS-SPME/GC-MS, in silico molecular docking, and in vitro bioactivity assays. This strategy allows for a comprehensive evaluation of the VOMs present in commonly used culinary herbs and spices, as well as their potential to modulate targets associated with neurodegenerative, oxidative, and inflammatory pathways. By bridging metabolomics with computational and experimental pharmacology, this study provides novel insights into the therapeutic potential of culinary herbs and spices, extending their relevance beyond traditional uses and offering a foundation for future nutraceutical development.

## 2. Materials and Methods

### 2.1. Chemicals

All chemicals and reagents used were of analytical grade. HPLC-grade methanol was obtained from Fischer Scientific (Loughborough, UK). Gallic acid (purity ≥ 99%), quercetin (≥98%), sodium chloride (NaCl, 99.5%), 3-octanol (internal standard, 99%), 6-hydroxy-2,5,7,8-tetramethylchromane-2-carboxylic acid (Trolox, 98.0%), hydrochloric acid (HCl, 37% *v*/*v*), fluorescein, sodium acetate (CH_3_COONa), 2,2′-azobis(2-methylpropionamidine) dihydrochloride (AAPH), egg albumin (≥99%), and anhydrous sodium carbonate (Na_2_CO_3_, 99.8%) were sourced from Sigma-Aldrich (St. Louis, MO, USA). Aluminum chloride (AlCl_3_) and potassium chloride (KCl, 99.5%) were purchased from Riedel-de Haën^®^ (Seelze, Germany). The Folin–Ciocalteu reagent (FR, 2 N), 1,1-diphenyl-2-picrylhydrazyl (DPPH· ≈ 90%) in its free radical form, 2,2′-azinobis-(3-ethylbenzothiazoline-6-sulfonic acid) radical cation (ABTS, 98.0%), potassium persulfate (99.0%), and an alkane series (C8 to C20, 40 mg/L in n-hexane) were supplied by Fluka (Buchs, Switzerland). The SPME fiber coated with divinylbenzene/carboxen/polydimethylsiloxane (DVB/CAR/PDMS) (50/30 µm), SPME holder for manual sampling, and glass vials were purchased from 2025 Merck KGaA (Darmstadt, Germany. Ultrapure water (18 MΩ cm) was provided by the Milli-Q water purification system (Millipore, Milford, MA, USA).

### 2.2. Samples

Six culinary herbs and six spices were freshly obtained from a local market in Funchal (Madeira Island, Portugal) in 2024, namely lemon verbena (*Aloysia citrodora*), chives (*Allium schoenoprasum* L.), basil (*Ocimum basilicum* L.), sage (*Salvia officinalis*), coriander (*Coriandrum sativum* L.), parsley (*Petroselinum crispum*), curcuma (*Curcuma longa* L.), nutmeg (*Myristica fragrans*), cumin (*Cuminum cyminum*), black pepper (*Piper nigrum* L.), Jamaica pepper (*Pimenta dioica* L.), and juniper berry (*Juniperus communis* L.). All samples were milled and homogenized using a grinder (A11 Basic analytical mill, IKA, Staufen, Germany) and stored at 25 ± 1 °C until analysis.

### 2.3. HS-SPME Procedure to Extract Volatile Organic Metabolites

HS-SPME extraction was carried out following the method described by Izcara et al. [21], with minor modifications. Briefly, 2 g of fresh sample, 0.5 g of NaCl, 5 mL of deionized water, and 5 µL of 3-octanol (102 µg/mL), along with a stirring bar, were added to a 20 mL glass vial, which was then sealed with a PTFE/silicone septum. The DVB/CAR/PDMS fiber was exposed to the vial’s headspace for 45 min at 40 ± 1 °C. After exposure, the fiber was retracted into the needle, and volatile organic metabolites (VOMs) were thermally desorbed at 250 °C for 6 min by introducing them into the GC injection port. Prior to use, the SPME fiber was thermally conditioned as per the manufacturer’s guidelines. Each analysis was performed in triplicate (*n* = 3), and a blank run was conducted between samples to ensure no carryover of analytes.

### 2.4. GC-MS Conditions

The volatile fingerprints of the culinary herbs and spices were analyzed using GC-MS. The analysis was conducted with an Agilent Technologies 6890N gas chromatograph connected to an Agilent 5975 quadrupole mass selective detector. A BP20 capillary column (60 m × 0.25 mm I.D. × 0.25 µm) was utilized for this analysis. The temperature program began at 40 °C and increased at a rate of 2.7 °C/min until reaching 220 °C, with a total run time of 73.67 min. Helium of 5.0 purity was used as the carrier gas at a constant flow rate of 1.3 mL/min to separate the analytes. Data acquisition was conducted over a mass range of 30–350 *m*/*z* using electron ionization at an energy level of 70 eV. VOMs were identified by comparing their GC retention times (RTs), Kovat index (KI), and mass spectra with those of available standards, as well as by matching their mass spectra with the National Institute of Standards and Technology (NIST) MS 05 spectral database (Gaithersburg, MD, USA) with a matching probability greater than 80%. Kovat index values were calculated using the van den Dool and Kratz equation and compared to values reported in the literature for similar columns [22]. The relative area was determined by adding 3-octanol (internal standard, IS) using the following equation: Relative area = (VOM GC peak area/IS GC peak area).

### 2.5. Molecular Modeling

#### 2.5.1. Preparation of Target Proteins

The crystal structure of the target proteins (receptors) were obtained through RCSB Protein Data Bank (https://www.rcsb.org/): AChE (code: 4PQE) with resolution 2.90 Å; BChE (code: 6ESY) with resolution 2.80 Å; MAO-B (code: 2V5Z) with resolution 1.60 Å; iNOS (code: 4NOS) with resolution 2.25 Å; 5-LOX (code: 6N2W) with resolution 2.71 Å; COX-2 (code: 5KIR) with resolution 2.70 Å. ChimeraX 1.2.5. [23] was used to verify and remove the presence of non-standard residues, and AutoDockTools-1.5.7 (ADT) [24] was used to convert the receptors from Protein Data Bank (PDB) to Protein Data Bank, partial charge (Q), and atom type (T) (PDBQT).

#### 2.5.2. Preparation of Small-Molecule Inhibitors

For each enzyme, their substrate and known inhibitors were chosen. Among them, the VOMs with VIPs higher than 1.5 and the most representative of culinary herbs and spices of each sample were selected for further investigation with docking studies. The molecules were drawn in Avogadro2 (version 1.91.0) [25]. Their energy was optimized using the force field general AMBER force field (GAFF), and the molecules were then converted to PDBQT with ADT. Figure S1 presents the structures of the compounds (ligands) used in this work.

#### 2.5.3. Molecular Docking Simulations

A series of docking simulations were conducted for six receptors, namely anti-Alzheimer’s (AChE, BChE), antioxidant (MAO-B, iNOS), and anti-inflammatory (5-LOX, COX-2). In addition, 5-LOX may also lead to antioxidant effects as it generates lipid peroxides. The grid boxes used in this study encompass the entire receptor. This approach was adopted to mitigate an experimental study in which the ligand would have access to the entire receptor and not just the active site. The molecule files were converted to PDBQT using ADT, and configuration files were created to provide commands to Vina. Finally, AutoDock Vina (version 1.2.3.) software [26,27] was used to generate the detailed ligand-receptor interactions, in which the binding affinity (ΔG) was expressed as kcal/mol. The results were analyzed using ChimeraX. All software used in the current study is open source.

### 2.6. In Vitro Assessment of Total Phenolic Content, Total Flavonoids Content, and Antioxidant and Anti-Inflammatory Activities of Culinary Herb and Spice Extracts

An aqueous solution was prepared to evaluate the total phenolic content (TPC), total flavonoid content (TFC), and total anthocyanin content (TAC), as well as the antioxidant and anti-inflammatory activities of the culinary herbs and spices being studied. All measurements were conducted in triplicate. Specifically, 2 g of the samples were boiled in 100 mL of hot water for 15 min. Following this, the mixture was centrifuged at 5000 rpm for 5 min (using a SIGMA 1–7 centrifuge, with a maximum capacity of 6 × 15 mL and a maximum relative centrifugal force of 6153× *g*). The supernatant was then collected and stored at −80 °C for subsequent in vitro assays.

#### 2.6.1. Total Phenolic Content

The TPC was measured spectrophotometrically using the Folin–Ciocalteu test as outlined by Abreu et al. [28]. A calibration curve was established with gallic acid as the reference standard, covering a concentration range of 15 to 76 mg/L, to quantify the TPC in culinary herb and spice extracts. The results were reported as milligrams of gallic acid equivalent per gram of sample [mg GAE/g]. Spectrophotometric readings were taken at 765 nm using a UV-Vis spectrophotometer (Lambda 25, Perkin Elmer, Waltham, MA, USA).

#### 2.6.2. Total Flavonoid Content

The TFC was determined using the AlCl_3_ colorimetric assay according to Abreu et al. [28]. In brief, 3 mL of the appropriately diluted extract and 3 mL of a 2% (*w*/*v*) AlCl3 solution in methanol were combined in a 10 mL screw-capped centrifuge tube and vortexed for 30 s. A slight yellow color developed, and the mixture was then incubated for 10 min in the dark at 25 ± 1 °C. The absorbance of all samples was measured at 300 nm using a spectrophotometer. Quercetin served as the reference standard for constructing a calibration curve within a concentration range of 5 to 25 mg/L. The results were reported as milligrams of quercetin equivalent per grams of sample [mg QE/g].

#### 2.6.3. Total Anthocyanin Content

The TAC of culinary herb and spice extracts was calculated using the pH differential assay reported by Ribeiro et al. [29]. This spectroscopic method involved measuring the absorbance of the extracts at 520 and 700 nm at two different pH levels: 1.0 (using KCl, 0.025 mol/L) and 4.5 (using CH3COONa, 0.40 mol/L). For the assay, 0.5 mL of the extract was placed in a 5 mL screw-capped centrifuge tube, resulting in two dilutions: one with pH 1.0 buffer and the other with pH 4.5 buffer. The absorbance values were then converted using a molar absorption coefficient of 26,900 L/mol·cm, and the results were expressed as total milligrams of cyanidin-3-glucoside per gram of sample, denoted as mg CGE/g. Water was used as the control.

#### 2.6.4. 2,2-Diphenyl-1-Picrylhydrazyl Scavenging Assay (DPPH)

The antioxidant activity of the DPPH^●^ free radical-scavenging capacity (A_AR_) was determined according to the procedure proposed by Abreu et al. [28]. Briefly, a stock solution of DPPH^●^ radical in methanol (400 μM) was prepared and maintained at 25 ± 1 °C in the dark. For the experiment, this stock solution was diluted in methanol to attain a working solution with an absorbance of 0.900 (±0.030) at 515 nm. A calibration curve was constructed using Trolox at concentrations ranging from 25 to 600 mg/L. Results were expressed as mg of Trolox equivalents (TEs) per g sample, mgTE/g. DPPH quenching assays were performed in triplicate. Methanol was used as a control.

#### 2.6.5. 2,2′-Azinobis-(3-Ethylbenzothiazoline-6-Sulfonic Acid) Scavenging Assay (ABTS)

The ABTS assay was performed according to Abreu et al. [28]. A 50 mL solution of 2,2′-azinobis-(3-ethylbenzothiazoline-6-sulfonic acid) radical cation (ABTS^+^) (20 mM) was prepared in phosphate-buffered saline (PBS pH 7.4), and 200 µL of potassium persulfate solution (70 mM) was added to the ABTS^●^ solution. The solution was stored at 25 ± 1 °C for 16 h to obtain a stable radical cation. After that, the solution was diluted in PBS to achieve a working solution with an absorbance of 0.900 (±0.030) at 734 nm. For the reaction, 12 µL of extract was added to 3 mL of the working solution and incubated in the dark for 20 min at 25 ± 1 °C. A calibration curve (100–1200 mg/L) was obtained by plotting the concentrations of Trolox against the percentage of inhibition of ABTS^●^. Results were expressed as mgTE/g. ABTS assays were performed in triplicate. PBS was used as a control.

#### 2.6.6. Oxygen Radical Absorbance Capacity (ORAC)

ORAC was conducted, as per Zulueta et al. [30], with slight modification. In a plate with 96 white flat-bottom wells, 50 µL of fluorescein (1.56 nM), 50 µL of extract, and 25 μL of AAPH (221 mM) were added. The plate was incubated at 37 °C for 15 min. The fluorescence was read at 485 nm and 520 nm (excitation and emission wavelength, respectively) in a Victor3 Multilabel Plate Counter 1420 fluorescence reader at 5 min intervals until the fluorescence decayed and the absorbance became constant. PBS and Trolox (20 µM) were used as the control and reference, respectively. The results were expressed in µmol (TE)/g.

#### 2.6.7. Anti-Inflammatory Activity

In vitro anti-inflammatory activity was assessed by measuring the inhibition of egg albumin denaturation, following the method described by Gunathilake et al. [31]. For the assay, 0.5 mL of 1% bovine serum albumin (BSA) in PBS at pH 6.4 was combined with 0.5 mL of the culinary herb and spice extracts in a 5 mL screw-capped centrifuge tube. The reaction mixture was vortexed for 30 s and then incubated at 37 °C for 15 min. It was subsequently heated at 70 °C for 5 min. After cooling, the turbidity was measured at 660 nm using a UV/VIS spectrophotometer, with PBS serving as the control. The results were expressed as the percentage inhibition of albumin denaturation.

### 2.7. Statistical Analysis

Statistical analysis was conducted using the MetaboAnalyst 6.0 web-based tool [32]. The raw GC-MS data and results from in vitro assays were pre-processed, normalized (through cubic root transformation and autoscaling), and then subjected to one-way analysis of variance (ANOVA), followed by Fisher’s test for post hoc multiple comparisons. A significance level of *p* < 0.05 was used to identify meaningful differences in the data from the culinary herbs and spices. Additionally, partial least squares-discriminant analysis (PLS-DA) was applied to the volatilomic fingerprint dataset of the culinary herbs and spices to analyze sample separations and pinpoint volatile organic metabolites (VOMs) that contribute to the differentiation of sample sets, focusing on those with variable importance in projection (VIP) scores greater than 1.5. Pearson correlation analysis was performed to examine the relationships among key VOMs and the in vitro assay results.

## 3. Results and Discussion

### 3.1. Volatilomic Fingerprint of Culinary Herbs and Spices

**Figure 1:**
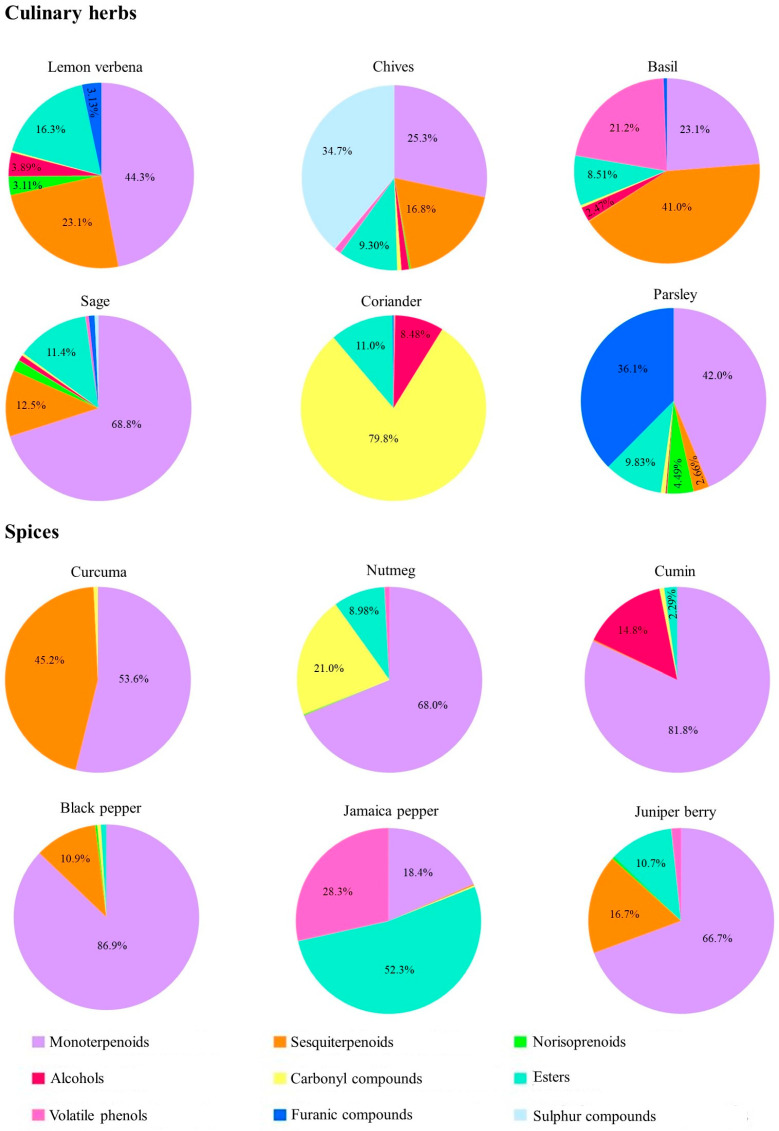
Contribution of each chemical family to the total volatilomic fingerprint of all culinary herbs and spices analyzed (for the portions of the pie whose percentage is not represented this means that the percentage value is less than 1%).

The volatilomic fingerprint of culinary herbs and spices was established using HS-SPME/GC-MS, a robust method for assessing authenticity and quality due to their nutritional value, high consumer demand, and distinctive flavors. In addition, HS-SPME/GC-MS offers several notable advantages over traditional extraction procedures. HS-SPME is a solvent-free, non-destructive technique that minimizes sample handling and preparation time while reducing the risk of analyte loss or degradation. This is particularly advantageous when working with volatile or semi-volatile VOMs, which are often difficult to recover efficiently using traditional procedures (e.g., liquid–liquid extraction, Soxhlet). Moreover, HS-SPME selectively enriches VOMs present in the headspace, minimizing matrix interferences and improving detection sensitivity. When coupled with GC-MS, this approach allows for high-resolution separation and reliable identification of VOMs, making it especially suitable for complex biological or food-related samples. Compared to traditional approaches, HS-SPME/GC-MS also aligns well with green chemistry principles by eliminating the need for organic solvents and reducing sample volume requirements. These features contribute to the robustness, efficiency, and environmental sustainability of the analytical workflow employed in this study. In total, 121 volatile organic metabolites (VOMs) were identified across the culinary herbs and spices analyzed, including forty-three monoterpenoids, thirteen sesquiterpenoids, four norisoprenoids, six alcohols, eighteen carbonyl compounds, eleven esters, four volatile phenols, three furanic compounds, eight sulfur compounds, and eleven other categories. Notably, limonene was the only VOM present in all samples examined. Additionally, as shown in Table S1, 14 VOMs were detected exclusively in certain species (such as α-thujene, 4-carene, perillene, β-terpineol, α-fenchol, hotrienol, (*E*)-piperitrol, and anethole), while 45 VOMs were unique to specific culinary herbs (including α-terpinene, o-cymene, α-ciclocitral, (+)-camphor, isoborneol, geranial, β-cubebene, aromadrene, calamine, nerolidol, cadalene, β-damascenone, geranylacetone, and β-ionone). Furthermore, sulfur compounds were identified exclusively in culinary herbs, predominantly in chives (Figure 1).

Table S1 presents the retention time, Kovat index, chemical family, and relative peak area of each VOM identified in the culinary herbs and spices. Monoterpenoids accounted for an average of 34.0 ± 0.99% in culinary herbs and 62.6 ± 1.31% in spices. Esters contributed 6.58 ± 1.27% in culinary herbs and 12.2 ± 1.80% in spices, while sesquiterpenoids made up 10.1 ± 2.21% in culinary herbs and 10.8 ± 3.27% in spices. Carbonyl compounds represented 14.9 ± 1.41% in culinary herbs and 3.47 ± 2.78% in spices. These chemical families had the largest impact on the volatilomic fingerprint of the culinary herbs and spices studied. Overall, the contribution of terpenoids and esters to the volatilomic fingerprint was nearly double in spices compared to culinary herbs, while carbonyl compounds had a contribution nearly four times higher in culinary herbs than in spices. Other chemical families contributed less than 6% on average to the total volatile profile.

#### 3.1.1. Culinary Herbs

Lemon verbena is a medicinal herb and a source of antioxidant compounds (e.g., VOMs, polyphenols). In total, 55 VOMs were identified in lemon verbena, with the majority being terpenoids (42.2%), followed by sesquiterpenoids (22.0%) and esters (20.2%). The most prevalent VOMs were β-caryophyllene, β-citronellol, phellandral, geranial, nonyl acetate, and (*Z*)-methyl isoeugenol, contributing to 47.9% of the total volatile profile. Kim and Lee [17] and Rashid et al. [12] reported fewer VOMs using HS-SPME/GC-MS, identifying only 14 and 27, respectively, which aligns with the current findings. Chives, known for their use in food seasoning and health benefits, had 38 identified VOMs, predominantly sulfur compounds (34.8%), monoterpenoids (25.3%), and sesquiterpenoids (16.8%), comprising 76.8% of the volatile profile. Key VOMs included dipropyl disulfide, dipropyl trisulfide, geranial, limonene, β-caryophyllene, and nerolidol, which accounted for 54.0% of the total volatile fingerprint. These results are consistent with earlier studies by Dai et al. [6] and Hanif et al. [33]. Basil, known for its medicinal uses, had 45 VOMs identified, primarily sesquiterpenoids (41.2%), monoterpenoids (23.0%), and volatile phenols (21.2%). The major VOMs were 3-allylguaiacol, β-cubebene, and geranial, contributing 27.9%, 20.1%, and 8.4% of the total volatile profile, respectively, in agreement with findings by Du et al. [34] and Mahmoud et al. [7]. Sage, another herb with healing properties, had 54 VOMs, mostly monoterpenoids (68.8%), esters (12.5%), and sesquiterpenoids (11.4%), making up 92.3% of its volatilomic fingerprint. The predominant VOMs, including isoborneol, (+)-camphor, β-thujone, eucalyptol, β-caryophyllene, and nonyl acetate, represented 65.7% of the total volatilomic fingerprint. These results align with those of Pachura et al. [10]. Coriander, used in both cooking and traditional medicine, had 37 VOMs identified, mostly carbonyl compounds (79.9%), esters (11.1%), and alcohols (8.40%). It is important to consider that cooking and other thermal processes can lead to substantial losses of VOMs due to their high vapor pressure and thermal instability, potentially reducing both their aromatic contribution and biological activity in the final preparation. The dominant VOMs in coriander included decanal, (*E*)-2-decenal, (*E*)-2-dodecenal, (*E*)-2-decen-1-ol, bornyl benzoate, and nonyl acetate, which made up 79.9% of the volatilomic fingerprint, consistent with Wei et al. [13]. Parsley, widely used in food and pharmaceuticals, had 23 VOMs identified, predominantly monoterpenoids (42.1%), furanic compounds (36.0%), and esters (9.84%). The main VOMs were o-cymene, 2-methyl-2,3-dihydrobenzofuran, and methyl salicylate, representing 72.2% of the volatilomic fingerprint.

#### 3.1.2. Spices

A total of 30 VOMs were identified in Curcuma, where the chemical families of monoterpenoids (53.6%) and sesquiterpenoids (45.3%) were the most abundant, representing 98.9% of their total volatile fingerprint. The most abundant VOMs in the analyzed curcuma were cuminaldehyde, τ-cadinol, and γ-curcumene, representing 42.4, 39.3, and 3.43% of the total volatile fingerprint, respectively. Qiang et al. [11] used the HS-SPME/GC-MS methodology to establish the volatilomic profile of curcuma essential oil and identified curcumene as one of the most predominant VOM. Nutmeg is a traditional spice commonly used worldwide. Myristicin, which is present in nutmeg, is responsible for its antioxidant, anti-inflammatory, and neuroprotective properties [9]. A total of 39 VOMs were tentatively identified, mainly characterized by monoterpenoids (68.1%), carbonyl compounds (20.9%), and esters (8.97%). Myristicin, γ-terpinene, hotrienol, and (*Z*)-methyl isoeugenol represented 20.5%, 12.4%, 7.94, and 6.87% of their total volatile fingerprints, respectively. Cumin, another popular spice, has been studied because of its favorable properties, including antioxidant, anti-inflammatory, antibacterial, and antidiabetic properties. A total of 33 VOMs have been identified in cumin. The total volatile fingerprint of this spice was highly influenced by monoterpenoids, representing 81.8% of the total volatile fingerprints. Alcohols and esters were also identified but contributed to a lesser extent to the volatile fingerprint of cumin (14.8% and 2.29%, respectively). Cuminaldehyde, γ-terpinene, β-pinene, and *p*-cymene accounted for 77.0% of the total volatile fingerprint. Twenty-nine VOMs were identified in black pepper, mainly monoterpenoids (86.9%) and sesquiterpenoids (10.9%), representing 97.8% of their total volatile fingerprints. The dominant VOMs identified in black pepper included 3-carene, β-thujone, limonene, 4-carene, and β-bourbonene, representing 82.0% of their total volatile fingerprints. Several VOMs identified in our study agree with the data provided in a previous study [8]. Because of its volatile fingerprint, Jamaica pepper is used in cooking and traditional medicine. The two major VOMs in Jamaica pepper are eugenol and methyl eugenol, and their content depends on the individual tree and the harvest time [5]. Forty-two VOMs were tentatively identified in Jamaica peppers. Esters (52.4%), volatile phenols (28.4%), and monoterpenoids (18.2%) are the most abundant chemical families detected in Jamaica pepper, representing 99.0% of the total volatile fingerprint. (*Z*)-Methyl isoeugenol and eugenol were the main VOMs identified in Jamaica pepper, accounting for 51.3% and 28.1% of the total volatile fingerprint, respectively. Thirty-five VOMs were identified in Juniper berry. Monoterpenoids (67.0%), sesquiterpenoids (16.5%), and esters (10.6%) are the main chemical families found in the juniper berry investigated, representing 94.1% of their total volatile fingerprint (Figure 1). This contribution is mainly provided by the β-myrcene, germacrene D, and sabinene, which account for 26.9%, 12.9%, and 15.1% of the total volatile fingerprint of juniper berry, respectively.

### 3.2. Statistical Analysis

**Figure 2:**
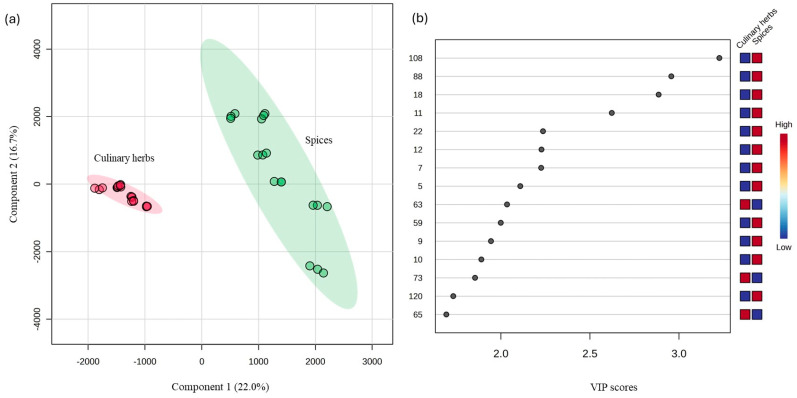
PLS-DA of the total volatilomic fingerprint of the culinary herbs and spices (*n* = 3 for each data point): (**a**) score scatter plot and (**b**) variable importance in projection (VIP) scores.

In the current study, the VOMs identified in culinary herbs and spices were analyzed using PLS-DA to explore the differences between the two groups. As illustrated in Figure 2a, the PLS-DA score plot demonstrates a clear distinction between culinary herbs and spices, indicating distinct VOM profiles. To identify the most influential VOM contributing to this distinction, VIP scores were examined (Figure 2b). VIP scores greater than 1.5 were considered significant, highlighting the key VOMs responsible for group discrimination. These included β-pinene (#5), 3-carene (#7), β-myrcene (#9), α-phellandrene (#10), 4-carene (#11), limonene (#12), γ-terpinene (#18), *p*-cymene (#22), hotrienol (#59), nonyl acetate (#63), β-caryophyllene (#65), isoborneol (#73), cuminaldehyde (#88), (*Z*)-methyl isoeugenol (#108), and τ-cadinol (#120). These VOMs are likely associated with the characteristic aroma profiles and phytochemical composition of each group, supporting their potential as chemical markers for the differentiation of culinary herbs and spices.

**Figure 3:**
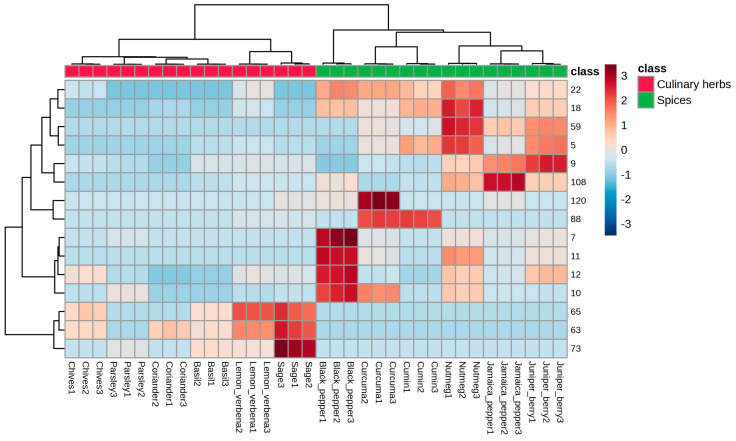
HCA and heatmap of the investigated culinary herbs and spices generated by the average algorithm and Pearson distance analysis.

Figure 3 presents the hierarchical cluster analysis (HCA) and heat map for the VOMs with VIP values greater than 1.5, using the average algorithm and Pearson distance analysis. This visualization highlights the dataset’s patterns, often used to identify samples or features with notably high or low values. The heatmap indicates that nonyl acetate (#63), β-caryophyllene (#65), and isoborneol (#73) were positively correlated with culinary herbs. In contrast, spices were strongly linked to VOMs such as β-pinene (#5), 3-carene (#7), β-myrcene (#9), α-phellandrene (#10), 4-carene (#11), limonene (#12), γ-terpinene (#18), *p*-cymene (#22), hotrienol (#59), cuminaldehyde (#88), (*Z*)-methyl isoeugenol (#108), and τ-cadinol (#120).

### 3.3. Molecular Docking Simulations

In silico molecular docking simulations were performed on targets related to Alzheimer’s disease, including enzymes associated with cholinergic dysfunction (e.g., AChE, butyrylcholinesterase (BChE)), oxidative stress (e.g., monoamine oxidase B (MAO-B), iNOS), and neuroinflammation (e.g., 5-LOX, COX-2). AChE and BChE are closely associated with Alzheimer’s disease as they play a key role in regulating acetylcholine levels in the body. Inhibiting these enzymes reversibly can raise acetylcholine levels in the brain, potentially easing some Alzheimer’s symptoms [35]. Meanwhile, MAO-B and iNOS are linked to oxidative stress. MAO-B breaks down neurotransmitters, resulting in the release of reactive oxygen species (ROS), which can lead to neuronal cell death. iNOS is responsible for producing reactive nitrogen species (RNS), further contributing to oxidative stress [36,37]. 5-LOX and COX-2 are associated with inflammation. 5-LOX and COX-2 are crucial enzymes involved in the inflammatory process. 5-LOX is responsible for producing leukotrienes from arachidonic acid, while COX-2 converts arachidonic acid into prostaglandins. Inhibiting 5-LOX has been associated with a decrease in amyloid-beta (Aβ) production and tau phosphorylation, key factors in Alzheimer’s disease [38,39]. Similarly, inhibiting COX-2 helps lower inflammation by reducing the production of prostaglandins, which are powerful mediators of immune and inflammatory responses [40]. In Alzheimer’s disease, COX-2 is overexpressed, contributing to tau phosphorylation and the formation of neurofibrillary tangles [41].

**Figure 4:**
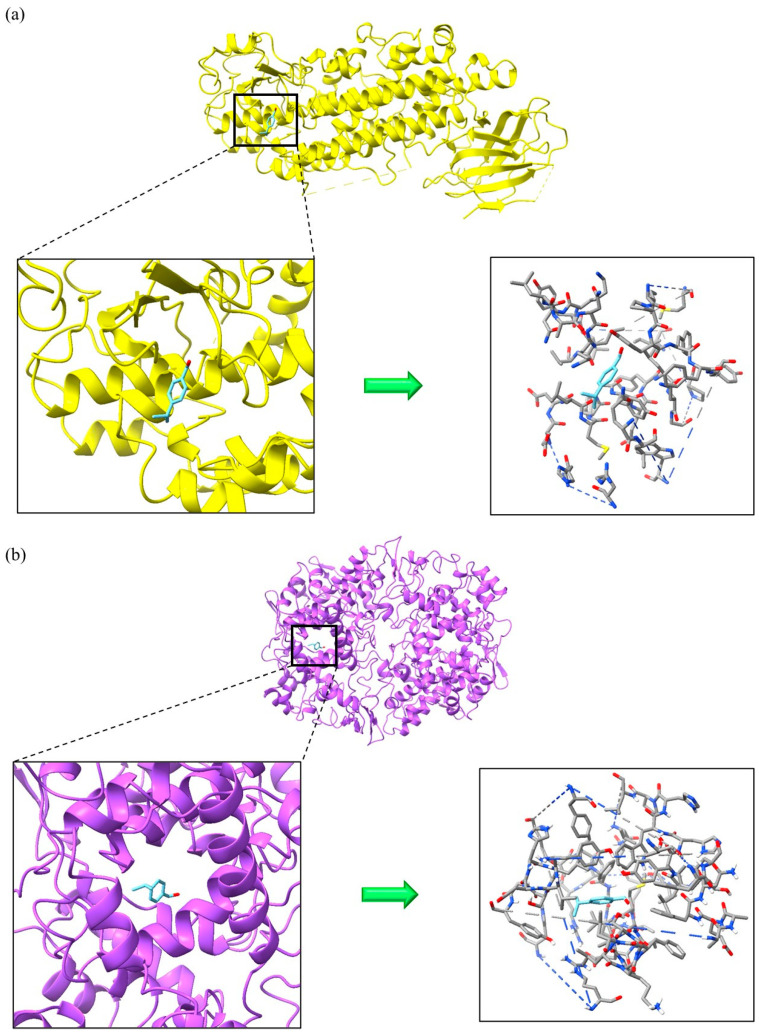
5-Lipoxygenase (**a**) and cyclooxygenase-2 (**b**) in complex with cuminaldehyde.

The primary VOMs found in culinary herbs and spices exhibited diverse binding energies for the targeted proteins (AChE, BChE, MAO-B, iNOS, 5-LOX, COX-2), as indicated by the Gibbs free energy (ΔG). Contemporary drug design protocols suggest that suitable candidates should have ΔG values below −6.0 kcal/mol. However, there is no agreed-upon range for the binding energies of physiologically active substances [42]. Table S2 reveals that cuminaldehyde, β-caryophyllene, γ-curcumene, germacrene D, and τ-cadinol displayed ΔG values under −6.0 kcal/mol for all examined receptors, suggesting their potential as anti-Alzheimer’s, antioxidant, and anti-inflammatory inhibitors. Nonetheless, these VOMs did not interact with COX-2 at docking scores lower than its inhibitors, specifically celecoxib (−8.666 kcal/mol) and meclofenamic acid (−8.477 kcal/mol). Additionally, as shown in Figure 4, cuminaldehyde interacted with 5-LOX at a docking score lower than nordihydroguaiaretic acid (−6.077 kcal/mol), while β-caryophyllene, γ-curcumene, germacrene D, and τ-cadinol exhibited docking scores lower than both nordihydroguaiaretic acid (−6.077 kcal/mol) and zileuton (−6.714 kcal/mol).

**Figure 5:**
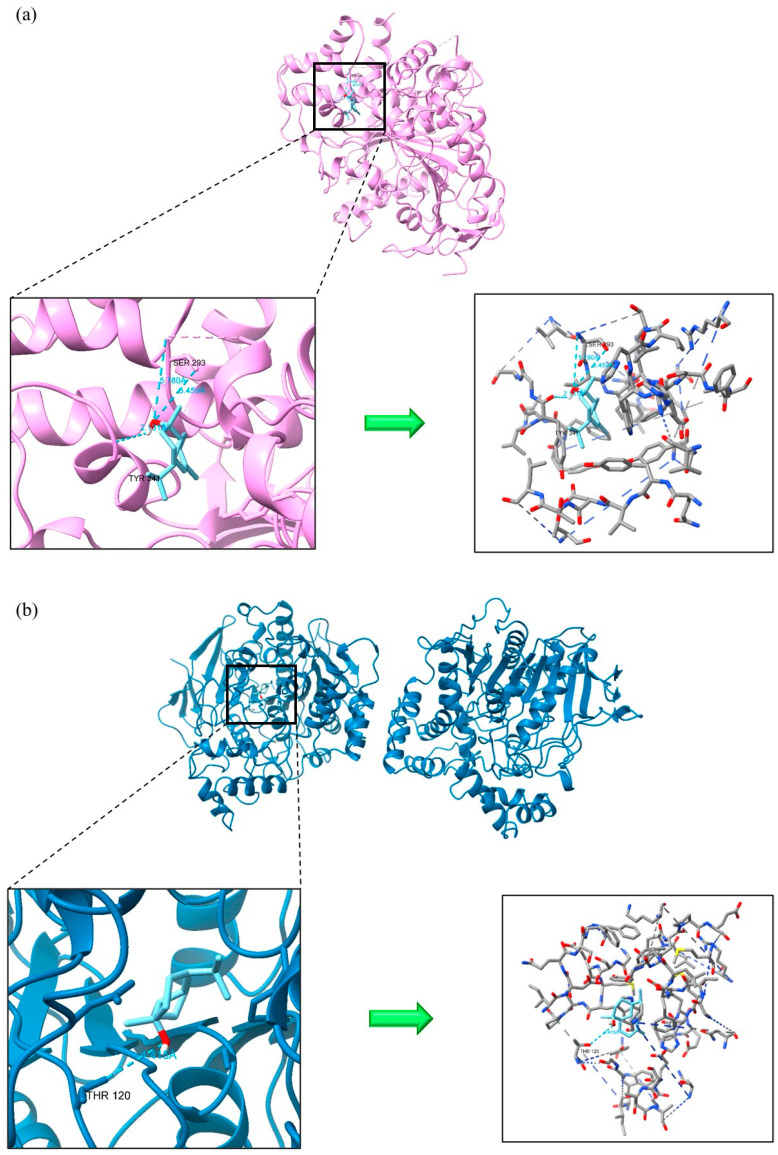
Acetylcholinesterase (**a**) and butyrylcholinesterase (**b**) in complex with τ-Cadinol.

**Figure 6:**
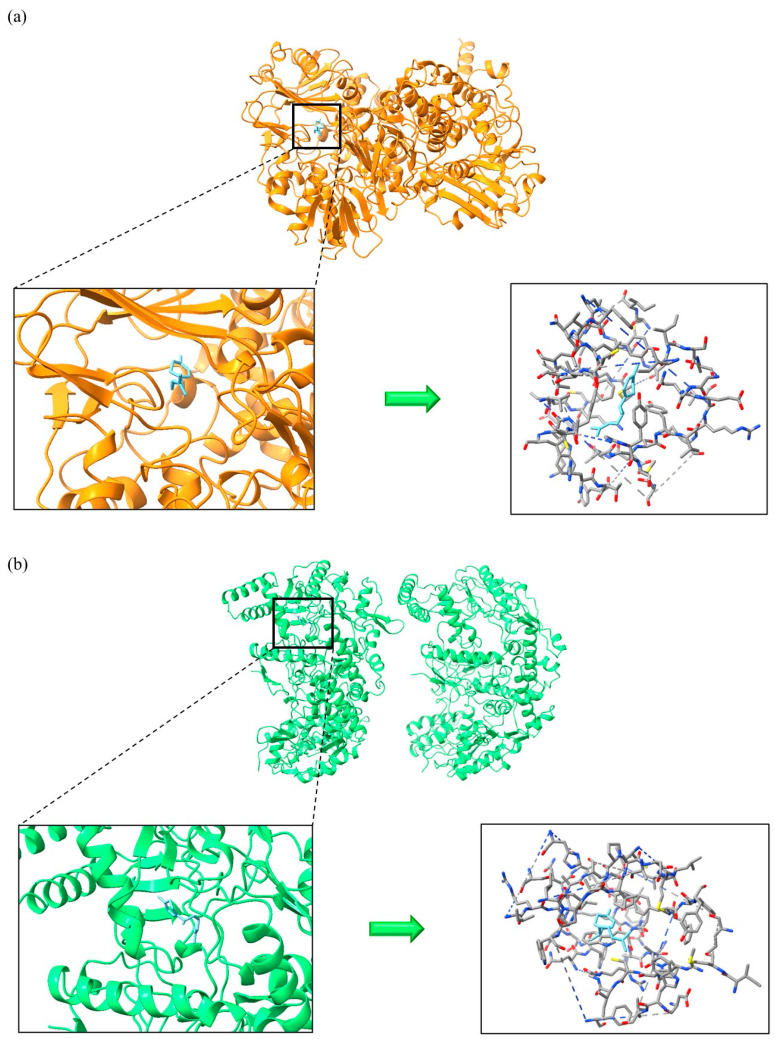
Monoamine oxidase B (**a**) and inducible nitric oxide synthase (**b**) in complex with γ-curcumene.

β-caryophyllene, germacrene D, and τ-cadinol exhibited stronger interactions with AChE and BChE compared to the inhibitor rivastigmine, as evidenced by their lower docking scores of −6.359 and −6.367 kcal/mol, respectively. τ-Cadinol formed multiple hydrogen bonds with AChE (three bonds: SER 293, TYR341) and BChE (one bond: THR 120), enhancing the stability of the complex, as shown in Figure 5. β-caryophyllene demonstrated superior interaction with AChE (−8.240 kcal/mol) compared to other inhibitors like donepezil (−7.439 kcal/mol), galantamine (−7.535 kcal/mol), and huperzine A (−7.557 kcal/mol). Regarding antioxidant receptors, γ-curcumene was the only compound to interact with MAO-B at a lower docking score (−8.208 kcal/mol) than the inhibitor safinamide (−8.125 kcal/mol), as illustrated in Figure 6. For iNOS, although there are no FDA-approved inhibitors, nitroarginine and 1400 W are commonly used despite lacking FDA approval, possibly due to efficacy or safety concerns [43]. Notably, cuminaldehyde (−7.220 kcal/mol), γ-curcumene (−7.975 kcal/mol), and τ-cadinol (−7.695 kcal/mol) showed stronger interactions with iNOS compared to the ΔG values of nitroarginine (−5.584 kcal/mol) and 1400W (−6.761 kcal/mol). For instance, β-caryophyllene and γ-curcumene, which showed high binding affinities in our molecular docking simulations, have been previously described as modulators of inflammatory pathways via NF-κB and COX-2 inhibition [44,45]. Similarly, cuminaldehyde has been associated with neuroprotective effects in rodent models of cognitive decline, supporting the relevance of our in silico predictions [46].

Conversely, although (*E*)-2-decen-1-ol, decanal, (*E*)-2-decenal, nonyl acetate, dipropyl disulfide, and dipropyl trisulfide were identified as primary VOMs in certain examined samples, the computational molecular docking simulations failed to indicate any potential for these VOMs to function as inhibitors for the receptors chosen in this investigation.

While the molecular docking simulations revealed promising interactions between certain VOMs (e.g., β-caryophyllene, germacrene D, τ-cadinol, γ-curcumene) and neurodegeneration-related targets (e.g., AChE, BChE, MAO-B), it is important to note that these findings are predictive. Docking results offer theoretical insights into binding affinity and potential mechanisms of action but do not confirm biological efficacy. Although the observed antioxidant and anti-inflammatory effects of the extracts support mechanisms often implicated in neuroprotection, direct evidence of anti-Alzheimer’s activity, such as inhibition of cholinesterase enzymes in vitro or neuroprotective effects in neuronal models, was not included in the scope of this study. Future work should aim to experimentally validate these computational predictions using functional assays relevant to Alzheimer’s disease pathology. Moreover, the complexity of culinary herb and spice extracts and potential synergistic or antagonistic interactions among VOMs were not explored in detail and may influence overall bioactivity.

### 3.4. Assessment of the Total Phenolics and Flavonoids and Antioxidant and Anti-Inflammatory Activities of Culinary Herbs and Spices

The antioxidant and anti-inflammatory activities of the culinary herb and spice aqueous extracts were analyzed through in vitro assays (TPC, TFC, TAC, DPPH, ABTS, ORAC, and inhibition of protein denaturation).

#### 3.4.1. Total Phenolic, Flavonoid, and Anthocyanin Content

The TPC of aqueous extracts of the culinary herbs and spices using the Folin–Ciocalteu assay is presented in Table 1. Folin–Ciocalteu assay measures the extract’s TPC based on its reducing capacity. Phenolics react with the Folin–Ciocalteu reagent (phosphomolybdenum/phosphotungsten complex) under alkaline conditions to form a blue complex, quantified spectrophotometrically. Jamaica pepper demonstrated the highest TPC level, 65.4 mgGAE/g DW, and lemon verbena the lowest, 2.50 mgGAE/g. On average, the TPC levels in spices (22.3 ± 0.45 mgGAE/g DW) are 4.37 times higher than those determined in culinary herbs (5.11 ± 0.15 mgGAE/g DW). No statistically significant differences (*p* < 0.05) in TPC levels were observed between chives, coriander, and curcuma, as well as between basil, sage, curcuma, and nutmeg. It is important to underscore that Section 3.1. assesses VOMs, mainly hydrophobic and low-molecular-weight compounds, whereas this section examines aqueous extracts rich in polar, water-soluble phytochemicals, such as polyphenols. These compositional differences likely contribute to the distinct biological responses observed. For example, curcumin, a key bioactive in curcuma, is absent from its volatile profile but present in aqueous extracts, which may influence the outcomes reported.

TFC was assessed using a colorimetric method involving complexation with AlCl_3_. Flavonoids form a stable complex with AlCl_3_, resulting in a measurable color change. Absorbance is recorded spectrophotometrically, and results are usually expressed as quercetin (QE) or rutin equivalents. The obtained values for TFC, conducted by the AlCl_3_ assay, ranged from 0.62 to 36.9 mg (QE)/g DW, with the lowest values found in nutmeg (0.62 mgQE/g) and curcuma (0.99 mgQE/g) and the highest values in Jamaica pepper (36.9 mgQE/g) and black pepper (13.1 mgQE/g DW). On average, the TPC levels in spices (10.9 ± 0.43 mgQE/g) are 4.91 times higher than those determined in culinary herbs (2.22 ± 0.08 mgQE/g). No statistically significant differences (*p* < 0.05) in TFC levels were observed between lemon verbena and basil, as well as among chives, sage, and cumin, and among coriander, parsley, and curcuma. In the literature, several values are reported for the TPC and TFC levels in culinary herbs and spices, most of them referring to hydroalcoholic, methanol, or ethanolic extracts [12,47]. The extraction of phenolic compounds, in general, is more efficient using alcohol and/or alcohol mixed with water as a solvent than water, as was demonstrated by others [12,48,49], since it promotes the degradation of lipid cell membranes and releases the bioactive compounds from plant cells. In the current study, hot water was used as a solvent to extract the phenolic compounds to simulate teas and infusions and mimic the consumption of spices when seasoning food. Numerous substances employed in seasoning, including terpenes, polyphenols, and other phytochemicals, are well-known for their antibacterial and antioxidant qualities. These substances can aid in preventing lipid oxidation and microbial spoiling during food preparation, which helps to maintain the food’s safety and preservation. Upon ingestion, these bioactive compounds are subjected to digestion and metabolism in the gastrointestinal tract. Several factors, such as their chemical structure, interactions with the gut bacteria, and the presence of other food components, influence their absorption, bioavailability, and subsequent biological effects. Certain molecules may maintain their activity and have positive systemic effects, such as cardioprotective or anti-inflammatory effects, whereas others may be quickly eliminated or change into metabolites with less or different activity. Therefore, the impact of these compounds extends beyond food preservation, with potential implications for human health that warrant further investigation. Considering aqueous extracts, Ferreira et al. [50] reported lower levels of TPC for parsley and chives, 2.10 and 0.64 mgGAE/g DW, respectively. On the other hand, higher levels of TFC for parsley and chives were reported by Ferreira et al. [50], 8.89 and 11.9 mgQE/g DW, respectively. For basil, higher TPC and TFC levels were reported by Muzolf-Panek et al. [48], who found 11.4 mgGAE/g DW and 3.25 mgQE/g DW, respectively. Kiani et al. [51] found lower levels of TPC for basil and coriander in aqueous extracts, 3.15 and 3.02 mgGAE/g, respectively. In nutmeg, lower TPC and TFC levels were reported by Muzolf-Panek et al. [48], 2.17 mgGAE/g DW and 0.59 mgQE/g DW, respectively.

TAC was quantified using the pH differential method, which exploits the structural transformation of anthocyanins at different pH levels. The difference in absorbance between pH 1.0 and pH 4.5 allows for the accurate estimation of monomeric anthocyanin content. The TAC levels of the culinary herbs and spices investigated ranged from 0.30 to 23.9 mgCGE/g DW, with the lower value corresponding to cumin and the higher value to curcuma. No statistically significant differences (*p* < 0.05) in TAC levels were observed between basil and juniper berry. On the other hand, the TAC levels of lemon verbena, chives, coriander, parsley, and black pepper were null in the current study (Table 1). This result is not in agreement with those reported in the literature for coriander and cumin since Tashtoush et al. [52] determined TAC levels in coriander and cumin using different solvents (ethanol, methanol, acetone) and temperatures (20, 40, and 60 °C), ranging from 0.03 to 0.19 mgCGE/g DW and from 0.02 to 0.13 mgCGE/g DW, respectively. These results suggest that hot water may not be the most suitable solvent to extract anthocyanins from culinary herbs and spices since the TAC levels in hot water were lower than reported earlier from ethanolic or hydroalcoholic extracts [53]. This might be related to changes in the polarity of solvents, extraction procedures, and other environmental factors (e.g., light exposure, temperature, and soil compositions).

#### 3.4.2. Antioxidant Activity

The antioxidant activity of the aqueous extracts of culinary herbs and spices was evaluated using three complementary assays: DPPH, ABTS, and ORAC. The DPPH assay measures the ability of antioxidants to donate hydrogen atoms or electrons to neutralize the stable DPPH radical, resulting in a color change that is quantifiable by spectrophotometry. Similarly, the ABTS assay assesses radical scavenging activity through the quenching of the ABTS^●^ radical cation, which also leads to a measurable decrease in absorbance. Both assays primarily reflect the capacity of antioxidants to act via single-electron transfer mechanisms. In contrast, the ORAC assay evaluates the ability of antioxidants to inhibit peroxyl radical-induced oxidation, relying on hydrogen atom transfer reactions. Together, these assays provide a broader understanding of the antioxidant potential of the samples. The lack of consensus stems from the fact that in vitro assays, such as DPPH, ABTS, or ORAC, each target specific antioxidant mechanisms (e.g., electron donation, radical scavenging, or reducing power), and different antioxidants in a complex matrix may behave differently across these assays. As a result, relying on a single assay can overlook the broad spectrum of antioxidant interactions, highlighting the importance of using a combination of assays for a more comprehensive evaluation of antioxidant activity in culinary herbs and spices [50,53]. Consequently, three in vitro assays, namely DPPH, ABTS, and ORAC, based on different principles were applied to attain more precise results (Table 1). It was possible to observe that independently of the in vitro assay, Jamaica pepper again had the highest antioxidant activity, and lemon verbena had the lowest.

In DPPH, the highest value was found in Jamaica pepper (87.3 mgTE/g), followed by cumin (25.5 mgTE/g), juniper berry (19.3 mgTE/g), sage (11.8 mgTE/g), nutmeg (11.2 mgTE/g), black pepper (10.5 mgTE/g), chives (6.78 mgTE/g), curcuma (6.14 mgTE/g), coriander (5.72 mgTE/g), parsley (5.70 mgTE/g), basil (4.79 mgTE/g), and lemon verbena (1.97 mgTE/g). The antioxidant activity for chives, basil, coriander, parsley, and curcuma did not present statistically significant differences (*p* < 0.05). In ABTS, Jamaica pepper again had the highest value, with 187 ± 4.87 mgTE/g, and lemon verbena had the lowest, with 5.85 ± 0.48 mgTE/g. Statistically significant differences (*p* < 0.05) were observed in lemon verbena, cumin, black pepper, Jamaica pepper, and juniper berry. Results obtained by Trifan et al. [54] revealed higher antioxidant activity for nutmeg using DPPH and ABTS, 49.1 and 66.2 mgTE/g, respectively. In ORAC, similarly to ABTS, Jamaica pepper and lemon verbena had the highest and the lowest values, 107 and 1.71 µMTE/g, respectively. No statistically significant differences (*p* < 0.05) in ORAC values were observed among basil, coriander, cumin, black pepper, sage, parsley, and nutmeg, as well as among chives, curcuma, and juniper berry (Table 1). Results obtained by Ferreira et al. [50] revealed lower and higher antioxidant activity for parsley (110 µMTE/g DW) and chives (31.49 µMTE/g DW) aqueous extracts, respectively.

#### 3.4.3. Anti-Inflammatory Activity

The anti-inflammatory activity of culinary herbs and spices was evaluated using the denaturation of egg albumin assay since it is a cost-effective and rapid screening method to assess anti-inflammatory potential based on protein stabilization. Protein denaturation is a common underlying mechanism in inflammatory processes, as denatured proteins can elicit autoimmune responses. This assay mimics the denaturation of proteins during inflammation and is often used as a preliminary in vitro model to evaluate anti-inflammatory agents. Albumin denaturation occurs when an external substance like acid, base, heat, or organic solvent destroys the tertiary and secondary structure of a protein. As a result, the denaturation of tissue proteins is recognized as an inflammatory marker. Table 1 shows the percentage of inhibition of culinary herbs and spices. The literature indicates that anti-inflammatory agents must suppress protein denaturation by at least 20% [55]. In this sense, lemon verbena (3.65%) and coriander (10.3%) cannot be considered potential anti-inflammatory agents since their protein denaturation was lower than 20%. Chives (32.2%) and basil (37.9%), as well as sage, parsley, curcuma, and nutmeg, showed an inhibition percentage of protein denaturation that was not significantly different (*p* < 0.05) when compared to the remaining extracts investigated. The inflammatory activity of the extracts can be attributed to the presence of VOMs, phenolic compounds, and flavonoids, among other bioactive compounds present in culinary herbs and spices. Related to VOMs (e.g., limonene, linalool, isoborneol, camphor, α-pinene, β-caryophyllene, (*Z*)-methyl isoeugenol, eugenol, myristicin), several studies highlight their potential therapeutic applications in treating inflammatory diseases due to their antioxidant properties [56,57]. Perhaps these VOMs show promise, but some adverse effects have been reported, such as cytotoxicity and allergic reactions associated with certain compounds (e.g., α-pinene, camphor, myristicin). This emphasizes the need for further research to determine safe concentrations for therapeutic use [57].

### 3.5. Pearson Correlation Between Main Volatile Organic Metabolites and Biological Activities

**Figure 7:**
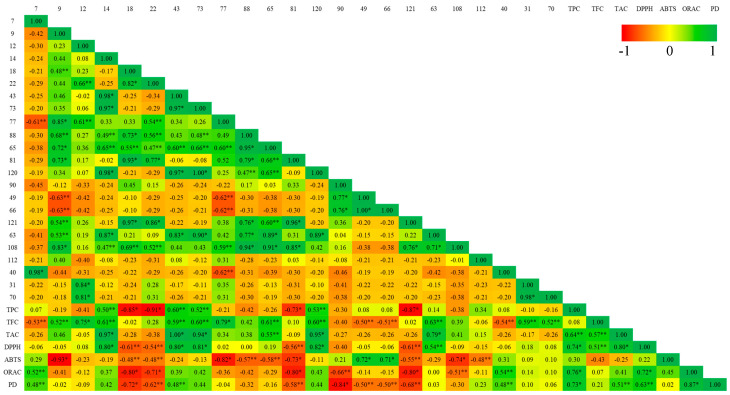
Pearson correlation between VOCs and bioactive activities of the culinary herbs (* significant difference at *p* < 0.001; ** significant difference at *p* < 0.05). Peak identification: 7—3-carene; 9—β-myrcene; 12—limonene; 14—eucalyptol; 18—γ-terpinene; 22—*p*-cymene; 43—β-thujone; 73—isoborneol; 77—geranial; 88—cuminaldehyde; 65—β-caryophyllene; 81—germacrene D; 120—τ-cadinol; 90—2-decen-1-ol; 49—decanal; 66—(*E*)-2-decenal; 121—myristicin; 63—nonyl acetate; 108—(*Z*)-methyl isoeugenol; 112—3-allylguaiacol; 40—2-methyl-2,3-dihydrobenzofuran; 31—dipropryl disulfide; 70—dipropyl trisulfide; Abbreviations: PD—egg albumin denaturation; TPC—total phenolic content; TFC—total flavonoid content; TAC—total anthocyanin content; ABTS—2,2′-azinobis-(3-ethylbenzothiazoline-6-sulfonic acid) scavenging assay; DPPH—2,2-diphenyl-1-picrylhydrazyl scavenging assay; ORAC—oxygen radical absorbance capacity.

**Figure 8:**
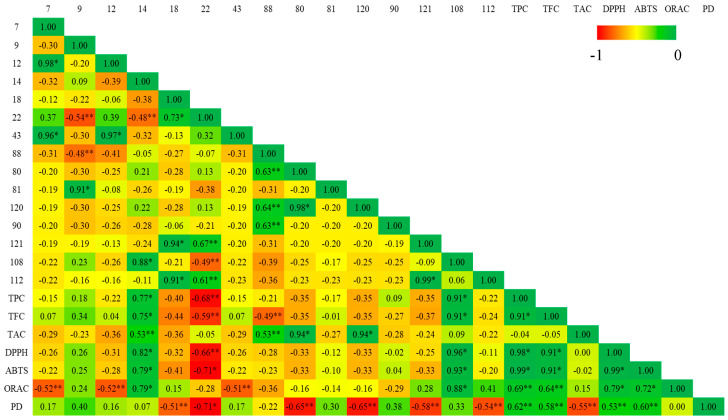
Pearson correlation between VOCs and bioactive activities of the spices (* significant difference at *p* < 0.001; ** significant difference at *p* < 0.05). Peak identification: 7—3-carene; 9—β-myrcene; 12—limonene; 14—eucalyptol; 18—γ-terpinene; 22—*p*-cymene; 43—β-thujone; 88—cuminaldehyde; 80—γ-curcumene; 81—germacrene D; 120—τ-cadinol; 90—(*E*)-2-decen-1-ol; 121—myristicin; 108—(*Z*)-methyl isoeugenol; 112—3-allylguaiacol; Abbreviations: PD—egg albumin denaturation; TPC—total phenolic content; TFC—total flavonoid content; TAC—total anthocyanin content; ABTS—2,2′-Azinobis-(3-ethylbenzothiazoline-6-sulfonic acid) scavenging assay; DPPH—2,2-diphenyl-1-picrylhydrazyl scavenging assay; ORAC—oxygen radical absorbance capacity.

To determine which primary VOMs in culinary herbs and spices might be linked to antioxidant and anti-inflammatory properties, a Pearson correlation analysis was conducted. The results of this analysis for various in vitro assays are displayed in Figure 7 and Figure 8 for herbs and spices, respectively. The strength and direction of the relationships were interpreted using the correlation coefficient (r). Positive correlations are indicated by values greater than zero, while negative correlations are shown by values less than zero. The strength of the correlation is categorized as follows: weak (0 to 0.3), moderate (0.3 to 0.7), and strong (0.7 to 1).

Analysis of culinary herbs revealed strong, statistically significant (*p* < 0.001) positive correlations between several antioxidant measures: TPC–DPPH (r = 0.74), TPC–ORAC (r = 0.76), TAC–DPPH (r = 0.80), and DPPH–ABTS (r = 0.72). Similarly, spices exhibited robust, significant (*p* < 0.001) positive correlations among various antioxidant parameters: TPC–TFC (r = 0.91), TPC–DPPH (r = 0.98), TPC–ABTS (r = 0.99), TFC–DPPH (r = 0.91), TFC–ABTS (r = 0.91), DPPH–ABTS (r = 0.99), DPPH–ORAC (r = 0.79), and ABTS–ORAC (r = 0.72). Regarding anti-inflammatory activities, spice extracts showed moderate, significant (*p* < 0.05) positive correlations between TPC-PD (r = 0.62), TFC-PD (r = 0.58), DPPH–PD (0.53), and ABTS–PD (r = 0.60). In contrast, culinary herbs demonstrated strong correlations between TPC–PD (r = 0.73) and ORAC-PD (r = 0.87, *p* < 0.001). These results indicate that the bioactive compounds in culinary herbs and spices may play a substantial role in their antioxidant and anti-inflammatory properties. Additionally, the antioxidant activity might be attributed to the chemical structure of these compounds, as well as their combined additive, synergistic, or antagonistic effects.

Regarding the main VOMs identified in spices, eucalyptol (#14) and (*Z*)-methyl isoeugenol (#108) showed a strong positive and significant (*p* < 0.001) correlation with TPC, TFC, DPPH, ABTS, and ORAC. γ-Curcumene (#80) and τ-cadinol (#120) showed a strong and significant (*p* < 0.001) correlation with TAC, while eucalyptol (#14) and cuminaldehyde (#88) showed a moderate correlation with TAC. On the other hand, for culinary herbs, eucalyptol (#14), β-thujone (#43), isoborneol (#73), and τ-cadinol (#120) showed a strong and significant (*p* < 0.001) correlation with TAC and DPPH assays. In addition, decanal (#49) and (*E*)-2-decenal (#66) strongly correlated with ABTS (*p* < 0.001). Eucalyptol (#14), β-thujone (#43), isoborneol (#73), and τ-cadinol (#120) demonstrated a moderate and significant (*p* < 0.001) correlation with TPC and TFC. β-caryophyllene (#65) and nonyl acetate (#63) showed a moderate and significant (*p* < 0.001) correlation with TFC and TAC. Regarding anti-inflammatory activities, a moderate positive and significant (*p* < 0.05) correlation was observed for the following main VOMs identified in culinary herbs: 3-carene (#7), β-thujone (#43), and 2-methyl-2,3-dihydrobenzofuran (#40). In spices, moderate correlation was observed without significance (Figure 8).

In addition, several VOMs showed a negative correlation with the egg albumin denaturation assay (Figure 7 and Figure 8), suggesting that higher levels of these metabolites may be associated with a reduced extent of detectable protein denaturation. This observation could be indicative of specific interactions between the metabolites and egg albumin that do not result in protein unfolding or precipitation. Instead, these VOMs may bind to albumin in a manner that stabilizes its native conformation or shields it from thermal or chemical stress. For example, curcumin, a major polyphenol from curcuma, has been shown to bind to human serum albumin via hydrophobic and hydrogen bonding interactions without inducing denaturation [58,59]. Similar binding mechanisms could be at play with the VOMs identified in our study. Such interactions may modulate the structural dynamics of albumin, potentially influencing its response in denaturation assays. Therefore, the negative correlations observed here should not be interpreted solely as a lack of activity but rather as indicative of a distinct mode of interaction, warranting further mechanistic studies.

## 4. Conclusions

The HS-SPME/GC-MS method successfully establishes the volatilomic fingerprint of culinary herbs and spices, revealing 121 VOMs. Limonene was the only VOM consistently present in all samples. The predominant chemical families were monoterpenoids (34.0 ± 0.99% in culinary herbs and 62.6 ± 1.31% in spices), esters (6.58 ± 1.27% in culinary herbs and 12.2 ± 1.80% in spices), sesquiterpenoids (10.1 ± 2.21% in culinary herbs and 10.8 ± 3.27% in spices), and carbonyl compounds (14.9 ± 1.41% in culinary herbs and 3.47 ± 2.78% in spices). In silico molecular docking, VOMs like cuminaldehyde, β-caryophyllene, γ-curcumene, germacrene D, and τ-cadinol exhibited ΔG values below −6.0 kcal/mol across multiple receptors, suggesting their potential as anti-Alzheimer’s, antioxidant, and anti-inflammatory agents. In vitro assays of aqueous extracts showed Jamaica pepper, juniper berry, black pepper, and cumin had the highest antioxidant and anti-inflammatory activities, while lemon verbena had the lowest. Notably, eucalyptol (#14) and (*Z*)-methyl isoeugenol (#108) showed strong correlations (*p* < 0.001) with various antioxidant metrics in spices, while eucalyptol (#14), β-thujone (#43), isoborneol (#73), and τ-cadinol (#120) were closely related to total antioxidant capacity and DPPH assays in culinary herbs. Additionally, 3-carene (#7), β-thujone (#43), and 2-methyl-2,3-dihydrobenzofuran (#40) were moderately correlated (*p* < 0.05) with anti-inflammatory activities.

These findings indicate that culinary herbs and spices are rich in bioactive metabolites that could be valuable for functional foods, nutraceuticals, and dietary supplements. They offer potential benefits for cognitive health, especially in preventing or delaying neurodegenerative diseases like Alzheimer’s. Their antioxidant and anti-inflammatory properties also make them suitable for cosmetic formulations aimed at protecting against oxidative stress and aging. Overall, the data obtained highlight the potential of culinary herbs and spices to modulate inflammatory processes, which may be relevant to diseases with an inflammatory component, such as Alzheimer’s and cardiometabolic diseases. Nevertheless, to confirm these potential effects, validation through in vivo studies and clinical trials is required. Future research should focus on experimentally validating the neuroprotective potential of key VOMs identified in this study, particularly cuminaldehyde, β-caryophyllene, and germacrene D. Investigating their mechanisms of action, bioavailability, and synergistic behavior in multi-compound systems will be crucial to determine their therapeutic relevance. Moreover, integrating metabolomic data with transcriptomic or proteomic approaches may offer deeper insight into the molecular pathways modulated by these culinary herb and spice extracts.

## Figures and Tables

**Table 1:** Total phenolic content (TPC), total flavonoid content (TFC), total anthocyanin content (TAC), antioxidant capacity (DPPH, ABTS, ORAC), and inhibition of protein denaturation of the aromatic herbs and spices. The values are expressed as mean ± SD per g of dry weight (DW).

Samples	TPC mgGAE/g	TFC mgQE/g	TAC mgC3GE/g	DPPH mgTE/g	ABTS mgTE/g	ORAC µMTE/g	Egg Albumin Denaturation (%)
Lemon verbena	2.50 ± 0.01 ^a^	2.28 ± 0.02 ^a^	n.d.	1.97 ± 0.05 ^a^	5.85 ± 0.48 ^a^	1.70 ± 0.09 ^a^	3.65 ± 0.17 ^a^
Chives	4.65 ± 0.12 ^b^	3.35 ± 0.20 ^a,b^	n.d.	6.78 ± 1.05 ^b^	20.7 ± 1.40 ^b^	63.9 ± 2.40 ^b^	32.2 ± 1.58 ^b^
Basil	6.06 ± 0.02 ^b,c^	2.02 ± 0.01 ^a,b^	0.70 ± 0.01 ^a^	4.79 ± 0.44 ^b^	7.78 ± 0.75 ^a,c^	48.1 ± 0.82 ^c^	37.9 ± 0.57 ^b^
Sage	6.73 ± 0.13 ^c^	3.49 ± 0.23 ^a,b^	2.06 ± 0.06 ^b^	11.8 ± 0.82 ^c^	15.9 ± 1.93 ^b,c^	87.1 ± 6.90 ^d^	47.3 ± 1.10 ^c^
Coriander	5.39 ± 0.36 ^b,c^	1.16 ± 0.05 ^a,c^	n.d.	5.72 ± 0.25 ^b^	34.8 ± 1.32 ^d^	46.7 ± 0.63 ^c^	10.3 ± 0.35 ^d^
Parsley	5.36 ± 0.24 ^b,c^	1.10 ± 0.01 ^a,c^	n.d.	5.70 ± 0.79 ^b^	25.4 ± 2.61 ^b,c^	94.4 ± 9.60 ^d,e^	48.9 ± 1.94 ^c^
Curcuma	6.16 ± 0.21 ^b,c^	0.99 ± 0.01 ^a,c^	23.9 ± 0.22 ^c^	6.14 ± 0.19 ^b^	35.4 ± 2.61 ^d^	62.7 ± 0.76 ^b^	45.5 ± 2.07 ^c^
Nutmeg	6.33 ± 0.10 ^c^	0.62 ± 0.03 ^c^	1.18 ± 0.06 ^d^	11.2 ± 0.08 ^c^	35.2 ± 4.77 ^d^	82.5 ± 0.37 ^d^	47.6 ± 4.54 ^c^
Cumin	26.4 ± 0.25 ^d^	3.31 ± 0.02 ^a,b^	0.31 ± 0.03 ^e^	25.5 ± 0.52 ^d^	79.7 ± 1.61 ^e^	56.5 ± 1.15 ^b,c^	77.0 ± 0.39 ^e^
Black pepper	15.3 ± 0.24 ^e^	13.1 ± 1.24 ^d^	n.d.	10.5 ± 1.12 ^c^	48.9 ± 2.79 ^f^	46.3 ± 0.60 ^c^	70.6 ± 0.35 ^f^
Jamaica pepper	65.4 ± 1.38 ^f^	36.9 ± 0.64 ^e^	8.45 ± 0.16 ^f^	87.3 ± 0.12 ^e^	187 ± 4.87 ^g^	107 ± 2.84 ^e^	76.6 ± 0.23 ^e^
Juniper berry	14.6 ± 0.50 ^e^	10.6 ± 0.64 ^d^	0.61 ± 0.02 ^a^	19.3 ± 1.10 ^f^	62.6 ± 3.66 ^h^	63.3 ± 2.94 ^b^	74.5 ± 0.37 ^e,f^

n.d.—not detected; GAE—gallic acid equivalents; QE—quercetin equivalents; C3G—cyanidin-3-glucoside equivalents; TE—Trolox equivalents. The same letter indicates no significant difference at *p* < 0.05.

## Data Availability

The original contributions presented in the study are included in the article/Supplementary Materials, and further inquiries can be directed to the corresponding authors.

## Supplementary Materials

The following supporting information can be downloaded at https://www.mdpi.com/article/10.3390/foods14091456/s1. Figure S1: Chemical structures of the volatile organic metabolites used in the molecular modeling study; Table S1: Relative peak area of volatile organic metabolites identified in culinary herbs and spices using HS-SPME/GC-MS; Table S2: Interacting residues, binding affinities (ΔG: kcal/mol), the number of hydrogen bonds (H-b), and residues H-bonding (Residues H-b) obtained through docking simulations.

## Abbreviations

The following abbreviations are used in this manuscript:

5-LOX	5-lipoxygenase
ABTS	2,2′-azinobis-(3-ethylbenzothiazoline-6-sulfonic acid)
AChE	acetylcholinesterase
ADT	AutoDockTools
Aβ	amyloid-beta
BChE	butyrylcholinesterase
C3GE	cyanidin-3-glucoside equivalents
COX-2	cyclooxygenase-2
CPO	cold-pressed oil
DPPH	1,1-diphenyl-2-picrylhydrazyl
DW	dry weight
FDA	Food and Drug Administration
GAE	gallic acid equivalent
GC-MS	gas chromatography–mass spectrometry
HCA	hierarchical cluster analysis
HS-SPME	headspace solid-phase microextraction
iNOS	inducible nitric oxide synthase
KI	Kovat index
MAO-B	monoamine oxidase B
ORAC	oxygen radical absorbance capacity
PBS	phosphate-buffered saline
PDB	Protein Data Bank
PDBQT	Protein Data Bank, partial charge, and atom type
PLS-DA	partial least squares-discriminant analysis
QE	quercetin equivalent
ROS	reactive oxygen species
RT	retention time
TAC	total anthocyanin content
TE	Trolox equivalents
TFC	total flavonoid content
TPC	total phenolic content
VIP	variable importance in projection
VOMs	volatile organic metabolites
ΔG	Gibbs free energy

## References

[1] Athanasiadis V., Chatzimitakos T., Makrygiannis I., Kalompatsios D., Bozinou E., Lalas S.I. (2024). Antioxidant-Rich Extracts from Lemon Verbena (*Aloysia citrodora* L.) Leaves through Response Surface Methodology. Oxygen.

[2] Bukvicki D., Gottardi D., Prasad S., Novakovic M., Marin P.D., Tyagi A.K. (2020). The Healing Effects of Spices in Chronic Diseases. Curr. Med. Chem..

[3] Şener G., Karakadıoglu G., Ozbeyli D., Ede S., Yanardag R., Sacan O., Aykac A. (2022). Petroselinum Crispum Extract Ameliorates Scopolamine-Induced Cognitive Dysfunction: Role on Apoptosis, Inflammation and Oxidative Stress. Food Sci. Hum. Wellness.

[4] Singh V., Kaur K., Kaur S., Shri R., Singh T.G., Singh M. (2022). Trimethoxyflavones from *Ocimum basilicum* L. Leaves Improve Long Term Memory in Mice by Modulating Multiple Pathways. J. Ethnopharmacol..

[5] Bajer T., Ligor M., Ligor T., Buszewski B. (2016). Design of the Extraction Process for Terpenes and Other Volatiles from Allspice by Solid-Phase Microextraction and Hydrodistillation. J. Sep. Sci..

[6] Dai X., Jia C., Lu J., Yu Z. (2023). The Dynamics of Bioactive Compounds and Their Contributions to the Antioxidant Activity of Postharvest Chive (*Allium schoenoprasum* L.). Food Res. Int..

[7] Mahmoud E., Starowicz M., Ciska E., Topolska J., Farouk A. (2022). Determination of Volatiles, Antioxidant Activity, and Polyphenol Content in the Postharvest Waste of *Ocimum basilicum* L. Food Chem..

[8] Milenković A., Stanojević J., Stanojević L. (2022). Comparative Analysis of Chemical Composition and Antioxidant Activity of Essential Oil and Hydrolate from Black Pepper Fruit (*Piper nigrum* L.). Maced. J. Chem. Chem. Eng..

[9] Muchtaridi, Subarnas A., Apriyantono A., Mustarichie R. (2010). Identification of Compounds in the Essential Oil of Nutmeg Seeds (*Myristica fragrans* Houtt.) That Inhibit Locomotor Activity in Mice. Int. J. Mol. Sci..

[10] Pachura N., Zimmer A., Grzywna K., Figiel A., Szumny A., Łyczko J. (2022). Chemical Investigation on *Salvia officinalis* L. Affected by Multiple Drying Techniques—The Comprehensive Analytical Approach (HS-SPME, GC–MS, LC-MS/MS, GC-O and NMR). Food Chem..

[11] Qiang Y., Si R., Tan S., Wei H., Huang B., Wu M., Shi M., Fang L., Fu J., Zeng S. (2021). Spatial Variation of Volatile Organic Compounds and Antioxidant Activity of Turmeric (*Curcuma longa* L.) Essential Oils Harvested from Four Provinces of China. Curr. Res. Food Sci..

[12] Rashid H.M., Mahmod A.I., Afifi F.U., Talib W.H. (2022). Antioxidant and Antiproliferation Activities of Lemon Verbena (*Aloysia citrodora*): An In Vitro and In Vivo Study. Plants.

[13] Wei S., Lyu J., Wei L., Xie B., Wei J., Zhang G., Li J., Gao C., Xiao X., Yu J. (2022). Chemometric Approaches for the Optimization of Headspace-Solid Phase Microextraction to Analyze Volatile Compounds in Coriander (*Coriandrum sativum* L.). LWT.

[14] Drinić Z., Pljevljakušić D., Janković T., Zdunić G., Bigović D., Šavikin K. (2021). Hydro-Distillation and Microwave-Assisted Distillation of Sideritis Raeseri: Comparison of the Composition of the Essential Oil, Hydrolat and Residual Water Extract. Sustain. Chem. Pharm..

[15] Palmieri S., Pellegrini M., Ricci A., Compagnone D., Lo Sterzo C. (2020). Chemical Composition and Antioxidant Activity of Thyme, Hemp and Coriander Extracts: A Comparison Study of Maceration, Soxhlet, UAE and RSLDE Techniques. Foods.

[16] Luca S.V., Kittl T., Minceva M. (2023). Supercritical CO2 Extraction of Spices: A Systematic Study with Focus on Terpenes and Piperamides from Black Pepper (*Piper nigrum* L.). Food Chem..

[17] Kim N., Lee D. (2004). Headspace Solid-Phase Microextraction for Characterization of Fragrances of Lemon Verbena (*Aloysia triphylla*) by Gas Chromatography-Mass Spectrometry. J. Sep. Sci..

[18] Baky M.H., Elkenawy N.M., El-Nashar H.A.S., Abib B., Farag M.A. (2024). Comparison of Autoclaving and γ-Radiation Impact on Four Spices Aroma Profiles and Microbial Load Using HS-SPME GC–MS and Chemometric Tools. Sci. Rep..

[19] Farag M.A., Dokalahy E.U., Eissa T.F., Kamal I.M., Zayed A. (2022). Chemometrics-Based Aroma Discrimination of 14 Egyptian Mango Fruits of Different Cultivars and Origins, and Their Response to -1 SPME Coupled to GC–MS. ACS Omega.

[20] Mostafa N.M., Mostafa A.M., Ashour M.L., Elhady S.S. (2021). Neuroprotective Effects of Black Pepper Cold-Pressed Oil on Scopolamine-Induced Oxidative Stress and Memory Impairment in Rats. Antioxidants.

[21] Izcara S., Perestrelo R., Morante-Zarcero S., Sierra I., Câmara J.S. (2022). Volatilomic Fingerprinting from Edible Flowers. Unravelling Some Impact Compounds behind Its Attractiveness. Food Biosci..

[22] El-Sayed A.M. The Pherobase: Database of Insect Pheromones and Semiochemicals. http://www.pherobase.com.

[23] Pettersen E.F., Goddard T.D., Huang C.C., Meng E.C., Couch G.S., Croll T.I., Morris J.H., Ferrin T.E. (2021). UCSF ChimeraX: Structure Visualization for Researchers, Educators, and Developers. Protein Sci..

[24] Sanner M.F. (1999). Python: A Programming Language for Software Integration and Development. J. Mol. Graph Model..

[25] Hanwell M.D., Curtis D.E., Lonie D.C., Vandermeersch T., Zurek E., Hutchison G.R. (2012). Avogadro: An Advanced Semantic Chemical Editor, Visualization, and Analysis Platform. J. Cheminform..

[26] Eberhardt J., Santos-Martins D., Tillack A.F., Forli S. (2021). AutoDock Vina 1.2.0: New Docking Methods, Expanded Force Field, and Python Bindings. J. Chem. Inf. Model..

[27] Trott O., Olson A.J. (2010). AutoDock Vina: Improving the Speed and Accuracy of Docking with a New Scoring Function, Efficient Optimization, and Multithreading. J. Comput. Chem..

[28] Abreu T., Jasmins G., Bettencourt C., Teixeira J., Câmara J.S., Perestrelo R. (2023). Tracing the Volatilomic Fingerprint of Grape Pomace as a Powerful Approach for Its Valorization. Curr. Res. Food Sci..

[29] Ribeiro L.F., Ribani R.H., Francisco T.M.G., Soares A.A., Pontarolo R., Haminiuk C.W.I. (2015). Profile of Bioactive Compounds from Grape Pomace (*Vitis vinifera* and *Vitis labrusca*) by Spectrophotometric, Chromatographic and Spectral Analyses. J. Chromatogr. B.

[30] Zulueta A., Esteve M.J., Frígola A. (2009). ORAC and TEAC Assays Comparison to Measure the Antioxidant Capacity of Food Products. Food Chem..

[31] Gunathilake K., Ranaweera K., Rupasinghe H. (2018). In Vitro Anti-Inflammatory Properties of Selected Green Leafy Vegetables. Biomedicines.

[32] Pang Z., Chong J., Zhou G., De Lima Morais D.A., Chang L., Barrette M., Gauthier C., Jacques P.É., Li S., Xia J. (2021). MetaboAnalyst 5.0: Narrowing the Gap between Raw Spectra and Functional Insights. Nucleic Acids Res..

[33] Hanif M., Xie B., Wei S., Li J., Gao C., Wang R., Ali S., Xiao X., Yu J., Al-Hashimi A. (2022). Characterization of the Volatile Profile from Six Different Varieties of Chinese Chives by HS-SPME/GC–MS Coupled with E. NOSE. J. King Saud Univ.-Sci..

[34] Du P., Yuan H., Chen Y., Zhou H., Zhang Y., Huang M., Jiangfang Y., Su R., Chen Q., Lai J. (2023). Identification of Key Aromatic Compounds in Basil (*Ocimum* L.) Using Sensory Evaluation, Metabolomics and Volatilomics Analysis. Metabolites.

[35] Peitzika S.-C., Pontiki E. (2023). A Review on Recent Approaches on Molecular Docking Studies of Novel Compounds Targeting Acetylcholinesterase in Alzheimer Disease. Molecules.

[36] Iova O.-M., Marin G.-E., Lazar I., Stanescu I., Dogaru G., Nicula C.A., Bulboacă A.E. (2023). Nitric Oxide/Nitric Oxide Synthase System in the Pathogenesis of Neurodegenerative Disorders—An Overview. Antioxidants.

[37] Jalil S., Basri R., Aziz M., Shafiq Z., Ejaz S.A., Hameed A., Iqbal J. (2024). Pristine 2-Chloroquinoline-Based-Thiosemicarbazones as Multitarget Agents against Alzheimer’s Disease: In Vitro and in Silico Studies of Monoamine Oxidase (MAO) and Cholinesterase (ChE) Inhibitors. J. Mol. Struct..

[38] Joshi Y.B., Praticò D. (2015). The 5-Lipoxygenase Pathway: Oxidative and Inflammatory Contributions to the Alzheimer’s Disease Phenotype. Front. Cell. Neurosci..

[39] Chu J., Praticò D. (2016). The 5-Lipoxygenase as Modulator of Alzheimer’s γ-Secretase and Therapeutic Target. Brain Res. Bull..

[40] Javed M.A., Bibi S., Jan M.S., Ikram M., Zaidi A., Farooq U., Sadiq A., Rashid U. (2022). Diclofenac Derivatives as Concomitant Inhibitors of Cholinesterase, Monoamine Oxidase, Cyclooxygenase-2 and 5-Lipoxygenase for the Treatment of Alzheimer’s Disease: Synthesis, Pharmacology, Toxicity and Docking Studies. RSC Adv..

[41] Moussa N., Dayoub N. (2023). Exploring the Role of COX-2 in Alzheimer’s Disease: Potential Therapeutic Implications of COX-2 Inhibitors. Saudi Pharm. J..

[42] Gligorić E., Igić R., Teofilović B., Grujić-Letić N. (2023). Phytochemical Screening of Ultrasonic Extracts of Salix Species and Molecular Docking Study of Salix-Derived Bioactive Compounds Targeting Pro-Inflammatory Cytokines. Int. J. Mol. Sci..

[43] Minhas R., Bansal Y., Bansal G. (2020). Inducible Nitric Oxide Synthase Inhibitors: A Comprehensive Update. Med. Res. Rev..

[44] Lu S., Tang L., Zhou L., Lai Y., Liu L., Duan Y. (2022). Study on the Multitarget Mechanism and Active Compounds of Essential Oil from Artemisia Argyi Treating Pressure Injuries Based on Network Pharmacology. Evid.-Based Complement. Altern. Med..

[45] Iorio R., Celenza G., Petricca S. (2022). Multi-Target Effects of ß-Caryophyllene and Carnosic Acid at the Crossroads of Mitochondrial Dysfunction and Neurodegeneration: From Oxidative Stress to Microglia-Mediated Neuroinflammation. Antioxidants.

[46] Omari Z., Kazunori S., Sabti M., Bejaoui M., Hafidi A., Gadhi C., Isoda H. (2021). Dietary Administration of Cumin-Derived Cuminaldehyde Induce Neuroprotective and Learning and Memory Enhancement Effects to Aging Mice. Aging.

[47] Francik S., Francik R., Sadowska U., Bystrowska B., Zawiślak A., Knapczyk A., Nzeyimana A. (2020). Identification of Phenolic Compounds and Determination of Antioxidant Activity in Extracts and Infusions of Salvia Leaves. Materials.

[48] Muzolf-Panek M., Stuper-Szablewska K. (2021). Comprehensive Study on the Antioxidant Capacity and Phenolic Profiles of Black Seed and Other Spices and Herbs: Effect of Solvent and Time of Extraction. J. Food Meas. Charact..

[49] Alcântara M.A., de Lima Brito Polari I., de Albuquerque Meireles B.R.L., de Lima A.E.A., da Silva Junior J.C., de Andrade Vieira É., dos Santos N.A., de Magalhães Cordeiro A.M.T. (2019). Effect of the Solvent Composition on the Profile of Phenolic Compounds Extracted from Chia Seeds. Food Chem..

[50] Ferreira F.S., de Oliveira V.S., Chávez D.W.H., Chaves D.S., Riger C.J., Sawaya A.C.H.F., Guizellini G.M., Sampaio G.R., Torres E.A.F.D.S., Saldanha T. (2022). Bioactive Compounds of Parsley (*Petroselinum crispum*), Chives (*Allium schoenoprasum* L) and Their Mixture (*Brazilian cheiro-verde*) as Promising Antioxidant and Anti-Cholesterol Oxidation Agents in a Food System. Food Res. Int..

[51] Kiani H.S., Ali B. (2023). Optimized Extraction of Polyphenols, LC-MS/MS, and GC-MS Identification of Metabolites from the Selected Medicinal Herbs, Their Antioxidant and Anti-Diabetic Potential. Preprints.

[52] Tashtoush S.H., Ereifej K.I., Feng H., Rababah T.M., Al-U’datt M.H., Gammoh S., Al-Rabadi G.J. (2016). Temperature and Acidified Solvent Effect on Total Anthocyanins and RP-HPLC Phenolic Acids Determination in Selected Spices. Food Nutr. Sci..

[53] Kozłowska M., Ścibisz I., Przybył J., Ziarno M., Żbikowska A., Majewska E. (2021). Phenolic Contents and Antioxidant Activity of Extracts of Selected Fresh and Dried Herbal Materials. Pol. J. Food Nutr. Sci..

[54] Trifan A., Zengin G., Korona-Glowniak I., Skalicka-Woźniak K., Luca S.V. (2023). Essential Oils and Sustainability: In Vitro Bioactivity Screening of *Myristica fragrans* Houtt. Post-Distillation By-Products. Plants.

[55] Bailey-Shaw Y.A., Williams L.A.D., Green C.E., Rodney S., Smith A.M. (2017). In-Vitro Evaluation of the Anti-Inflammatory Potential of Selected Jamaican Plant Extracts Using the Bovine Serum Albumin Protein Denaturation Assay. Int. J. Pharm. Sci. Rev. Res..

[56] Oliveira S.D.D.S., De Oliveira E Silva A.M., Blank A.F., Nogueira P.C.D.L., Nizio D.A.D.C., Almeida-Pereira C.S., Pereira R.O., Menezes-Sá T.S.A., Santana M.H.D.S., Arrigoni-Blank M.D.F. (2021). Radical Scavenging Activity of the Essential Oils from *Croton grewioides* Baill Accessions and the Major Compounds Eugenol, Methyl Eugenol and Methyl Chavicol. J. Essent. Oil Res..

[57] Kim T., Song B., Cho K.S., Lee I.-S. (2020). Therapeutic Potential of Volatile Terpenes and Terpenoids from Forests for Inflammatory Diseases. Int. J. Mol. Sci..

[58] Kudva A.K., Manoj M.N., Swamy B.M., Ramadoss C.S. (2011). Complexation of Amphotericin B and Curcumin with Serum Albumins: Solubility and Effect on Erythrocyte Membrane Damage. J. Exp. Pharmacol..

[59] Behjati Hosseini S., Asadzadeh-Lotfabad M., Erfani M., Babayan-Mashhadi F., Mokaberi P., Amiri-Tehranizadeh Z., Saberi M.R., Chamani J. (2022). A Novel Vision into the Binding Behavior of Curcumin with Human Serum Albumin-Holo Transferrin Complex: Molecular Dynamic Simulation and Multi-Spectroscopic Perspectives. J. Biomol. Struct. Dyn..

