# Supplementary material for: Multifaceted Biological Activities of Culinary Herb and Spice Extracts: In Vitro and In Silico Simulation Insights into Inflammation-Related Targets
Source: Foods. 2025 Apr 23;14(9):1456. doi: 10.3390/foods14091456 (PMC12072151; doi:10.3390/foods14091456)
Supplement: Supplementary file 1 [file foods-14-01456-s001.zip › foods-3564674-supplementary.pdf]

Multifaceted biological activities of culinary herbs and spices extracts on Alzheimer's Disease prevention, focusing on antioxidant, anti-inflammatory and *in silico* analysis

Nance Hontman [1], Jéssica Gonçalves [1], José S. Câmara [1,2], Rosa Perestrelo [1,*]

[1] CQM – Centro de Química da Madeira, Universidade da Madeira, Campus da Penteada, 9020-105 Funchal, Portugal

[2] Departamento de Química, Faculdade de Ciências Exatas e Engenharia, Universidade da Madeira, Campus da Penteada, 9020-105 Funchal

* Corresponding authors: Tel.: (+351) 291705254; fax: (+351) 291705149.

E-mail address: rmp@staff.uma.pt

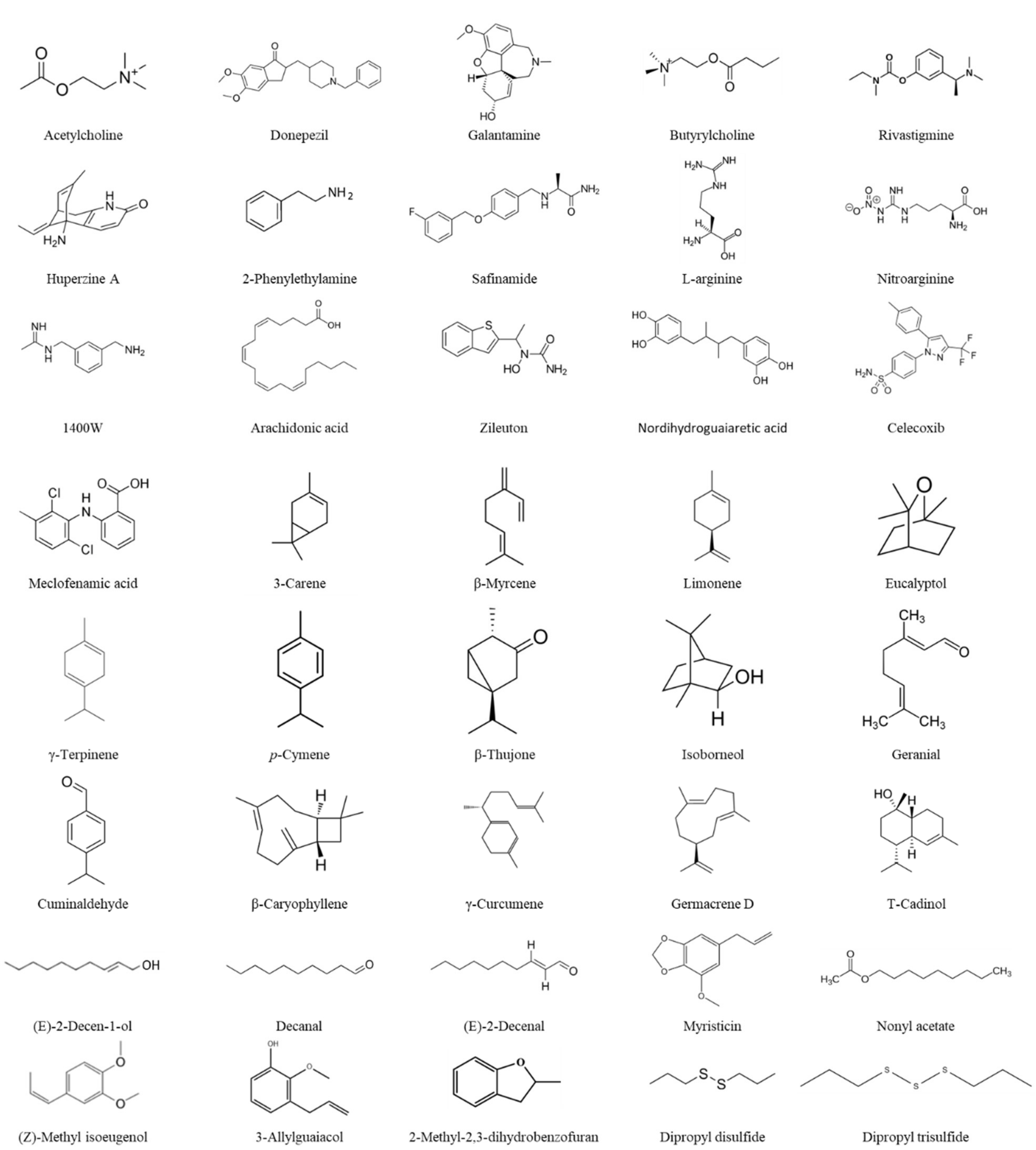

Figure S1. Chemical structures of the compounds used in the molecular modelling study.

Table S1. Relative peak area of volatile organic metabolites identified in culinary herbs and spices using HS-SPME/GC-MS.

RT (min)[a]	Peak Nº	KI cal[b]	KI lit[c]	Volatile organic metabolites	Culinary herbs						Spices					
					Lemon verbena	Chives	Basil	Sage	Coriander	Parsley	Curcuma	Nutmeg	Cumin	Black pepper	Jamaica pepper	Juniper berry
				**Monoterpenoids**												
17.23	1	1013	1013	α-Pinene	1.81 ± 0.19	0.43 ± 0.04	0.07 ± 0.01	0.09 ± 0.02	0.12 ± 0.01	0.74 ± 0.05	16.0 ± 0.63	-	5.67 ± 0.14	454 ± 33.6	8.75 ± 1.66	8.52 ± 0.46
17.88	2	1028	1021	α-Thujene	-	-	-	-	-	-	7.47 ± 0.40	121 ± 7.39	3.87 ± 0.37	3.19 ± 0.42	6.57 ± 0.64	-
19.01	3	1052	1052	Camphene	-	-	-	0.57 ± 0.07	-	-	-	16.5 ± 2.61	-	8.63 ± 1.02	0.76 ± 0.14	35.2 ± 6.42
20.95	5	1090	1090	β-Pinene	2.14 ± 0.19	-	0.13 ± 0.01	-	-	-	11.7 ± 0.73	321 ± 33.1	90.5 ± 14.6	-	8.52 ± 0.44	210 ± 17.8
21.29	6	1096	1096	Sabinene	5.20 ± 0.48	0.84 ± 0.11	-	-	-	7.36 ± 0.63	-	28.9 ± 4.02	7.25 ± 1.18	21.2 ± 0.55	9.07 ± 0.36	742 ± 21.0
22.24	7	1115	1114	3-Carene	-	-	-	-	-	2.21 ± 0.32	5.55 ± 0.68	17.3 ± 2.38	-	1499 ± 210	4.23 ± 0.32	12.3 ± 1.90
22.76	9	1126	1128	β-Myrcene	14.5 ± 1.29	2.70 ± 0.46	1.43 ± 0.04	0.99 ± 0.18	0.22 ± 0.03	2.97 ± 0.48	8.91 ± 1.25	119 ± 19.9	4.88 ± 0.39	-	523 ± 33.3	1325 ± 125
22.97	10	1130	1140	α-Phellandrene	1.30 ± 0.19	0.93 ± 0.15	-	0.18 ± 0.03	-	12.0 ± 0.92	189 ± 10.3	61.1 ± 5.88	2.73 ± 0.16	325 ± 49.9	5.65 ± 0.86	4.69 ± 0.52
23.71	11	1145	1149	4-Carene	-	-	-	-	-	-	12.0 ± 1.91	303 ± 32.9	-	865 ± 58.7	5.56 ± 0.54	25.6 ± 0.87
24.49	12	1159	1155	Limonene	41.6 ± 4.55	35.5 ± 2.86	0.18 ± 0.01	2.47 ± 0.39	0.44 ± 0.01	8.76 ± 1.10	21.5 ± 2.72	251 ± 30.8	5.44 ± 0.21	1350 ± 114	36.4 ± 3.89	375 ± 33.2
24.71	13	1163	1163	β-Phellandrene	2.34 ± 0.31	-	0.20 ± 0.01	-	-	6.28 ± 0.04	-	-	-	-	15.6 ± 2.15	38.1 ± 3.31
25.17	14	1172	1173	Eucalyptol	13.9 ± 0.79	2.58 ± 0.30	1.76 ± 0.03	12.9 ± 2.35	-	-	59.1 ± 9.71	10.7 ± 1.64	5.30 ± 0.78	-	154 ± 3.59	9.45 ± 1.27
26.59	17	1197	1195	β-Ocimene	1.09 ± 0.09	-	1.65 ± 0.11	-	0.37 ± 0.07	-	-	146 ± 18.2	6.28 ± 1.16	-	4.81 ± 0.22	1.82 ± 0.02
26.86	18	1201	1200	γ-Terpinene	4.28 ±0.69	-	-	-	0.52 ± 0.04	-	20.9 ± 1.73	618 ± 119	127 ± 9.61	56.8 ± 4.16	10.1 ± 1.10	76.3 ± 4.18
27.67	20	1218	1220	p-Menth-4(8)-ene	-	-	0.30 ± 0.04	0.12 ± 0.01	-	-	-	-	-	-	78.0 ± 4.62	-
27.90	21	1222	1225	α-Terpinene	-	-	-	-	0.44 ± 0.06	9.11 ± 0.24	-	-	-	-	-	-
28.40	22	1232	1232	p-Cymene	24.2 ± 3.42	6.59 ± 1.21	-	-	-	-	150 ± 4.88	262 ± 40.7	93.9 ± 18.2	120 ± 15.7	40.2 ± 4.23	76.1 ± 4.73
28.57	23	1235	1248	o-Cymene	13.2 ± 0.68	-	-	1.24 ± 0.16	0.43 ± 0.05	170 ± 16.0	-	-	-	-	-	-
30.53	25	1272	1284	δ-Terpinene	46.5 ± 8.71	-	-	0.23 ± 0.04	0.31 ± 0.03	23.3 ± 0.48	15.8 ± 2.66	172 ± 33.9	3.71 ± 0.11	33.3 ± 2.31	15.8 ± 1.82	69.0 ± 1.99
33.64	29	1330	1330	Alloocimene	0.64 ± 0.05	-	-	0.32 ± 0.04	-	-	1.86 ± 0.08	2.37 ± 0.30	-	1.30 ± 0.04	1.25 ± 0.17	43.4 ± 0.46
35.92	34	1374	1383	Fenchone	-	-	-	-	-	18.3 ± 1.79	-	-	-	1.14 ± 0.18	-	5.86 ± 0.38
36.58	36	1387	1405	Perillene	-	-	-	-	-	-	-	-	-	-	1.40 ± 0.22	6.22 ± 0.37
37.11	39	1396	1420	α-Cyclocitral	0.97 ± 0.06	31.2 ± 3.64	-	-	-	-	-	-	-	-	-	-
38.27	42	1421	1421	α-Thujone	-	-	-	9.71 ± 1.56	-	-	-	-	-	-	-	4.68 ± 0.52
38.47	43	1425	1425	β-Thujone	7.09 ± 0.34	-	5.71 ± 0.33	17.6 ± 2.15	-	-	-	-	-	1162 ± 185	-	-

38.93	44	1435	1437	β-Terpineol	-	-	-	-	-	-	-	38.3 ± 4.14	0.62 ± 0.04	1.52 ± 0.12	-	27.6 ± 0.72
42.18	52	1503	1501	(+)-Camphor	-	-	-	25.6 ± 2.19	-	-	-	-	-	-	-	-
42.34	53	1506	1506	Linalool	-	-	1.49 ± 0.11	1.07 ± 0.06	-	-	-	14.8 ± 2.24	1.35 ± 0.18	-	19.7 ± 0.79	14.4 ± 0.14
43.48	55	1531	1542	Limonene oxide	8.72 ± 0.98	-	-	-	-	-	13.9 ± 0.77	10.2 ± 1.27	-	-	1.73 ± 0.23	-
44.47	56	1552	1555	*α*-Fenchol	-	-	-	-	-	-	-	-	63.1 ± 10.3	-	-	-
45.57	59	1575	1573	Hotrienol	-	-	-	-	-	-	11.2 ± 1.80	396 ± 34.9	5.34 ± 0.99	-	49.8 ± 5.73	165 ± 5.35
48.52	68	1641	1649	Estragole	52.1 ± 4.50	-	3.53 ± 0.29	1.75 ± 0.31	-	-	-	-	-	25.4 ± 1.08	48.3 ± 7.07	-
49.55	72	1665	1665	*α*-Terpineol	64.1 ± 5.71	-	-	1.63 ± 0.22	-	-	-	32.0 ± 5.89	-	-	-	-
49.62	73	1666	1664	Isoborneol	13.6 ± 0.82	-	2.24 ± 0.38	68.8 ± 0.10	-	6.26 ± 0.47	-	-	-	-	-	-
51.03	76	1698	1700	Phellandral	147 ± 9.01	-	-	-	-	-	-	-	17.3 ± 1.90	-	-	-
51.18	77	1701	1706	Geranial	126 ± 16.4	56.4 ± 3.86	13.5 ± 0.80	9.20 ± 1.23	-	-	-	-	-	-	-	-
51.39	78	1707	1705	(*E*)-Piperitol	-	-	-	-	-	-	-	12.6 ± 0.82	-	-	-	-
53.53	87	1759	1756	β-Citronellol	157 ± 14.1	-	4.83 ± 0.35	9.49 ± 1.16	-	-	46.8 ± 5.84	-	-	-	3.23 ± 0.31	-
54.04	88	1771	1771	Cuminaldehyde	8.24 ± 0.38	-	-	0.50 ± 0.08	-	-	2360 ± 199	-	2439 ± 162	-	-	-
55.21	93	1799	1798	Anethole	-	-	-	-	-	-	4.87 ± 0.35	4.61 ± 0.21	-	-	1.90 ± 0.35	19.7 ± 0.99
55.61	94	1808	1809	p-Cymen-8-ol	8.21 ± 0.45	-	-	0.34 ± 0.06	-	-	8.09 ± 0.29	4.09 ± 0.30	-	6.34 ± 0.77	3.26 ± 0.57	-
56.93	99	1838	1842	Geraniol	-	-	0.09 ± 0.01	-	-	-	13.2 ± 1.99	430 ± 37.0	24.4 ± 1.50	7.12 ± 0.48	-	-
69.11	116	2155	2173	Carvacrol	-	5.81 ± 0.79	-	6.81 ± 1.06	-	-	-	5.90 ± 1.06	12.3 ± 0.58	-	-	-
				**Sesquiterpenoids**												
42.76	54	1515	1529	β-Bourbonene	10.8 ± 0.71	6.91 ± 0.96	-	0.61 ± 0.08	-	3.09 ± 0.51	-	-	-	730 ± 91.6	-	-
44.49	57	1553	1558	β-Cubebene	10.1 ± 0.19	-	45.0 ± 7.67	-	-	-	-	-	-	-	-	-
45.77	60	1579	1579	*α*-Bergamotene	-	-	-	6.23 ± 0.85	-	-	21.2 ± 1.62	-	-	-	-	-
46.09	62	1586	1589	Aromandrene	-	-	2.28 ± 0.40	0.90 ± 0.16	-	-	-	-	-	-	-	167 ± 19.7
47.84	65	1625	1623	β-Caryophyllene	203 ± 13.8	20.8 ± 1.71	3.28 ± 0.04	16.4 ± 1.15	-	-	-	-	-	-	-	-
49.00	69	1652	1653	β-Farnesene	44.9 ± 2.84	15.1 ± 1.68	1.53 ± 0.28	-	-	-	74.1 ± 13.5	-	8.93 ± 0.37	-	-	-
51.68	80	1714	1704	*γ*-Curcumene	-	-	-	-	-	-	239 ± 10.1	-	-	-	-	-
51.90	81	1719	1718	Germacrene D	53.0 ± 1.96	-	1.72 ± 0.05	0.78 ± 0.05	-	-	-	-	-	-	-	637 ± 22.2
55.98	95	1816	1816	Calamenene	3.95 ± 0.22	-	7.43 ± 0.19	-	-	-	-	-	-	-	-	-
59.31	104	1889	1901	*α*-Calacorene	45.0 ± 2.66	10.2 ± 1.89	2.33 ± 0.20	1.27 ± 0.09	-	7.99 ± 0.18	-	2.54 ± 0.21	-	4.91 ± 0.03	-	8.16 ± 0.58

60.30	107	2010	2008	Nerolidol	8.49 ± 0.56	35.7 ± 5.37	0.21 ± 0.01	0.19 ± 0.03	1.79 ± 0.18	-	-	-	-	-	-	-
70.22	118	2177	2188	Cadalene	21.1 ± 2.85	6.44 ± 0.91	2.60 ± 0.30	1.37 ± 0.14	-	5.85 ± 0.65	-	-	-	-	-	-
71.48	120	2186	2187	τ-cadinol	-	-	-	0.58 ± 0.03	-	-	2184 ± 280	-	-	10.4 ± 1.27	12.4 ± 2.19	-
				**Norisoprenoids**												
54.83	91	1790	1797	β-Damascenone	4.01 ± 0.24	1.73 ± 0.23	-	0.68 ± 0.11	-	-	-	-	-	-	-	-
56.13	96	1820	1825	*α*-Ionene	38.2 ± 3.09	-	-	3.94 ± 0.72	-	28.5 ± 2.45	-	8.79 ± 0.63	-	24.2 ± 2.18	-	19.9 ± 0.39
56.19	97	1821	1820	Geranylacetone	-	-	-	0.33 ± 0.06	-	-	-	-	-	-	-	-
59.65	105	1896	1896	β-Ionone	11.7 ± 2.11	-	0.16 ± 0.02	-	-	-	-	-	-	-	-	-
				**Alcohols**												
34.87	30	1354	1354	(*Z*)-3-Hexen-1-ol	-	-	-	-	5.90 ± 0.32	1.25 ± 0.12	-	-	-	-	-	-
35.84	33	1373	1373	(*E*)-2-Hexen-1-ol	3.87 ± 0.61	-	0.44 ± 0.02	0.46 ± 0.06	1.08 ± 0.07	-	-	-	-	-	-	-
49.96	74	1674	1692	(*E*)-2-Nonen-1-ol	-	-	-	-	1.77 ± 0.23	-	-	-	-	-	-	-
54.49	90	1782	1792	(E)-2-Decen-1-ol	51.1 ± 2.93	2.67 ± 0.45	2.88 ± 0.16	1.10 ± 0.06	77.6 ± 9.16	-	-	-	528 ± 46.9	-	-	-
58.09	102	1863	1863	Phenylethyl alcohol	4.28 ± 0.38	1.56 ± 0.26	0.42 ± 0.06	0.15 ± 0.03	13.9 ± 1.27	-	-	-	-	-	2.25 ± 0.23	-
62.32	109	2063	2076	1-Tridecanol	8.10 ± 1.35	2.35 ± 0.33	0.23 ± 0.04	0.67 ± 0.07	15.1 ± 0.95	-	-	-	-	-	-	-
				**Carbonyl compounds**												
26.36	16	1193	1192	2-Hexenal	-	-	-	-	6.29 ± 0.51	-	-	-	-	-	-	-
31.82	27	1294	1291	2-Heptenal	-	-	-	-	-	-	-	-	1.04 ± 0.19	-	1.51 ± 0.26	-
32.72	28	1312	1312	6-Methyl-5-hepten-2-one	0.62 ± 0.05	-	-	-	-	-	2.40 ± 0.07	1.10 ± 0.21	-	-	-	1.46 ± 0.10
35.52	32	1367	1367	Nonanal	-	-	-	-	1.21 ± 0.08	-	-	-	-	-	-	-
36.29	35	1381	1388	3-Octen-2-one	-	-	-	-	-	-	0.84 ± 0.04	-	1.62 ± 0.01	-	-	-
41.11	49	1481	1480	Decanal	-	-	-	0.26 ± 0.05	434 ± 41.2	2.77 ± 0.07	-	-	-	-	-	-
41.87	51	1496	1496	Benzaldehyde	-	-	0.33 ± 0.01	-	0.67 ± 0.06	-	6.03 ± 0.15	-	4.30 ± 0.53	-	4.04 ± 0.73	-
45.90	61	1582	1583	Undecanal	-	-	-	-	33.9 ± 5.49	-	-	-	-	-	-	-
47.88	66	1626	1628	(*E*)-2-Decenal	-	-	-	-	443 ± 50.3	2.33 ± 0.14	-	-	-	-	-	-
50.76	75	1692	1695	Dodecanal	-	-	-	-	11.1 ± 1.23	-	-	-	-	-	-	-
52.51	84	1734	1737	(*Z*)-7-Dodecenal	-	-	-	-	2.18 ± 0.20	-	-	-	-	-	-	-
52.58	85	1736	1743	(*E*,*Z*)-2,4-Decadienal	-	-	-	-	1.78 ± 0.22	-	-	-	-	-	-	-

55.12	92	1797	1794	Tridecanal	-	-	-	-	3.58 ± 0.31	-	-	-	-	-	-	-
57.00	100	1839	1830	(*E*)-2-Dodecenal	-	-	-	-	133 ± 17.5	-	-	-	-	-	-	-
59.22	103	1887	1888	(*Z*)-Cinnamaldehyde	2.37 ± 0.28	-	0.36 ± 0.04	0.11 ± 0.02	2.41 ± 0.27	-	32.7 ± 1.54	-	6.52 ± 0.57	8.35 ± 1.07	-	-
63.27	110	2087	2084	(*E*)-Cinnamaldehyde	-	3.67 ± 0.30	-	0.37 ± 0.05	3.76 ± 0.44	-	-	21.2 ± 3.03	4.02 ± 0.30	6.70 ± 1.18	-	-
70.07	117	2165	2171	Piperonal	-	-	-	0.27 ± 0.05	19.8 ± 0.20	-	-	-	-	27.5 ± 2.88	9.89 ± 1.49	-
71.55	121	2180	2205	Myristicin	1.96 ± 0.12	-	-	-	-	-	-	1021 ± 76.5	12.0 ± 1.67	-	-	-
				**Esters**												
31.47	26	1288	1290	(*Z*)-3-Hexen-1-ol acetate	-	-	-	-	4.40 ± 0.45	2.85 ± 0.49	-	-	-	-	-	-
39.18	46	1441	1454	Octyl acetate	-	-	0.40 ± 0.02	-	-	-	-	-	-	-	3.20 ± 0.35	-
45.01	58	1563	1554	Isobornyl acetate	63.6 ± 8.35	28.6 ± 1.10	3.64 ± 0.42	6.45 ± 0.71	-	-	-	21.6 ± 2.02	3.79 ± 0.12	-	-	33.2 ± 1.46
46.25	63	1589	1581	Nonyl acetate	145 ± 7.42	21.3 ± 2.05	4.08 ± 0.48	22.6 ± 1.22	63.1 ± 7.39	-	-	-	-	-	-	-
49.20	71	1657	1650	Decyl acetate	-	-	-	-	2.02 ± 0.24	-	-	-	-	-	-	-
51.66	79	1713	1722	9-Decenyl acetate	-	-	-	-	2.77 ± 0.31	-	-	-	-	-	-	-
52.22	83	1727	1728	Geranyl acetate	54.7 ± 3.38	-	-	0.83 ± 0.02	-	-	-	23.7 ± 2.87	-	28.6 ± 2.44	4.43 ± 0.77	295 ± 40.9
53.08	86	1748	1745	Methyl salicylate	-	-	-	-	-	59.7 ± 1.64	-	-	-	-	12.4 ± 1.26	-
62.16	108	2059	2059	(*Z*)-Methyl isoeugenol	91.1 ± 0.91	-	0.26 ± 0.03	0.37 ± 0.03	-	-	-	343 ± 53.9	4.80 ± 0.60	37.6 ± 7.10	2976 ± 416	194 ± 4.60
65.13	111	2115	2114	Bornyl benzoate	0.99 ± 0.08	2.44 ± 0.33	0.93 ± 0.08	0.50 ± 0.05	79.6 ± 9.08	-	-	1.75 ± 0.06	68.6 ± 3.48	-	-	-
68.05	113	2145	2126	(*E*)-Methyl isoeugenol	11.0 ± 1.11	-	4.31 ± 0.58	0.34 ± 0.02	-	-	-	57.4 ± 4.27	4.10 ± 0.19	-	39.0 ± 6.11	-
				**Volatile phenols**												
59.83	106	1947	1959	o-Cresol	-	-	0.08 ± 0.01	-	-	-	-	-	-	-	-	-
67.51	112	2140	-	3-Allylguaiacol	1.03 ± 0.07	-	32.4 ± 1.24	1.33 ± 0.06	-	-	-	41.9 ± 3.33	-	-	8.05 ± 1.28	-
68.93	115	2154	2158	Eugenol	-	5.85 ± 0.76	0.30 ± 0.05	-	-	-	-	-	-	-	1626 ± 282	79.0 ± 2.23
70.59	119	2170	2183	(*E*)-Isoeugenol	-	-	1.30 ± 0.20	-	-	-	-	-	-	-	13.8 ± 2.53	-
				**Furanic compounds**												
37.90	40	1413	-	2-Methyl-2,3-[illegible]	-	-	-	-	1.60 ± 0.23	229 ± 3.13	-	-	-	-	-	-
38.96	45	1436	1436	2-Furfural	-	-	-	-	0.57 ± 0.06	-	-	-	-	-	-	-
47.47	64	1616	1613	2-Furanmethanol	54.3 ± 2.43	-	0.88 ± 0.04	2.43 ± 0.07	-	-	-	-	-	-	2.25 ± 0.25	-
				**Sulphur compounds**												

22.50	8	1121	-	Allyl isopropyl sulfide	-	2.42 ± 0.37	-	-	-	-	-	-	-	-	-	-
25.42	15	1176	1181	2,4-Dimethylthiophene	-	1.00 ± 0.02	-	-	-	-	-	-	-	-	-	-
27.57	19	1216	1218	Methyl propyl disulfide,	-	2.08 ± 0.05	-	-	-	-	-	-	-	-	-	-
28.66	24	1237	1240	3,4-Dimethylthiophene	-	18.7 ± 1.98	-	-	-	-	-	-	-	-	-	-
35.31	31	1363	1365	Dipropyl disulfide	-	82.8 ± 7.32	-	1.69 ± 0.23	-	-	-	-	-	-	-	-
49.08	70	1654	1662	Dipropyl trisulfide	-	74.2 ± 13.6	-	-	-	-	-	-	-	-	-	-
52.09	82	1724	1723	2-Vinyl-1,3-dithiane	-	8.27 ± 1.22	-	-	-	-	-	-	-	-	-	-
54.34	89	1779	1775	cis-3,5-Diethyl-1,2,4-trithiolane	-	7.70 ± 0.98	-	-	-	-	-	-	-	-	-	-
				**Others**												
20.57	4	1083	1100	Undecane	-	-	-	-	2.60 ± 0.25	-	-	-	-	-	-	-
37.00	37	1394	-	3-Aminorhodanine	-	22.6 ± 2.11	-	-	-	-	-	-	-	-	-	-
37.03	38	1395	1394	α-p-Dimethylstyrene	54.3 ± 4.74	-	1.52 ± 0.28	0.94 ± 0.14	-	-	16.7 ± 1.41	17.4 ± 2.90	3.77 ± 0.22	13.2 ± 1.09	6.80 ± 0.98	10.2 ± 0.81
38.06	41	1417	1417	Acetic acid	-	-	-	-	-	-	-	-	-	3.35 ± 0.36	17.4 ± 2.38	7.79 ± 1.22
39.49	47	1447	1449	Tetramethyl pyrazine	-	-	-	-	-	-	5.37 ± 0.49	-	-	-	-	-
39.73	48	1452	-	2,4-Quinolinediol	-	-	-	-	-	-	-	14.8 ± 2.86	-	-	-	173 ± 3.57
41.63	50	1492	-	*m/z* 105, 119, 161	42.5 ± 2.52	22.3 ± 3.72	2.49 ± 0.09	3.37 ± 0.45	-	25.0 ± 1.56	-	-	-	-	-	-
47.97	67	1628	-	2,6-Dimethyl-2,6-octadiene	-	-	-	-	-	-	-	15.7 ± 1.80	-	-	-	-
56.50	98	1828	-	Propanethioamide	-	3.50 ± 0.32	-	-	-	-	-	-	-	-	-	-
57.42	101	1848	-	4-Ethyl-m-xylene	5.07 ±0.95	5.53 ± 0.88	0.20 ± 0.02	0.21 ± 0.02	-	-	-	-	-	-	-	-
68.39	114	2148	-	Iproniazid	-	6.59 ± 0.92	-	-	-	-	-	-	-	-	-	-

-: Not detected. [a] RT: Retention time. [b] Kovat index relative n-alkanes (C8 to C20) on a BP20 capillary column. [c] Kovat index relative reported in literature for equivalent capillary column (El-Sayed, 2011).

Table S2. Interacting residues, binding affinities (ΔG: kcal/mol), the number of hydrogen bonds (H-b), and residues H-bonding (Residues H-b) obtained through docking simulations.

	**Anti-Alzheimer receptors**						**Antioxidants receptors**						**Anti-inflammatory receptors**					
	**AChE**			**BChE**			**MAO-B**			**iNOS**			**5-LOX**			**COX-2**		
**Substrate**	**ΔG**	**H-b**	**Res. H-b**	**ΔG**	**H-b**	**Res. H-b**	**ΔG**	**H-b**	**Res. H-b**	**ΔG**	**H-b**	**Res. H-b**	**ΔG**	**H-b**	**Res. H-b**	**ΔG**	**H-b**	**Res. H-b**
Acetylcholine	-3.634	1	HIS 405	-	-	-	-	-	-	-	-	-	-	-	-	-	-	-
Butyrylcholine	-	-	-	-4.033	1	TYR 128	-	-	-	-	-	-	-	-	-	-	-	-
2-Phenylethylamine	-	-	-	-	-	-	-6.266	2	ALA 263<br>TYR 393	-	-	-	-	-	-	-	-	-
L-arginine	-	-	-	-	-	-	-	-	-	-6.564	10	CYS 15<br>CYS 110<br>CYS 115<br>GLY 117<br>SER 118<br>ALA 459<br>TRP 461<br>MET 480	-	-	-	-	-	-
Arachidonic acid	-	-	-	-	-	-	-	-	-	-	-	-	-4.494	6	ARG 370<br>SER 447<br>THR 545	-5.425	0	-
**Inhibitor**																		
Donepezil	-7.439	0	-	-8.835	1	THR 120	-	-	-	-	-	-	-	-	-	-	-	-
Galantamine	-7.535	5	TYR 124<br>SER 293<br>PHE 295	-8.230	3	ALA 199<br>TYR 440	-	-	-	-	-	-	-	-	-	-	-	-
Rivastigmine	-6.359	1	TYR 341	-6.367	1	THR 120	-	-	-	-	-	-	-	-	-	-	-	-

Huperzine A	-7.557	4	ASP 74<br>TYR 124<br>TRP 286<br>PHE 295	-8.960	7	ASP 70<br>THR 120<br>GLU 197<br>SER 198<br>ALA 199	-	-	-	-	-	-	-	-	-	-	-	-
Safinamide	-	-	-	-	-	-	-8.125	1	GLY 434	-	-	-	-	-	-	-	-	-
Nitroarginine	-	-	-	-	-	-	-	-	-	-5.584	7	SER 118<br>ILE 201<br>THR 376<br>ARG 381<br>TRP 461<br>ILE 462	-	-	-	-	-	-
1400W	-	-	-	-	-	-	-	-	-	-6.761	1	PRO 350	-	-	-	-	-	-
Zileuton	-	-	-	-	-	-	-	-	-	-	-	-	-6.714	3	HIS 225<br>GLN 656<br>LEU 657	-	-	-
Nordihydroguaiaretic acid	-	-	-	-	-	-	-	-	-	-	-	-	-6.077	6	HIS 360<br>GLN 363<br>THR 364<br>HIS 550	-	-	-
Celecoxib	-	-	-	-	-	-	-	-	-	-	-	-	-	-	-	-8.666	9	ARG 61<br>SER 121<br>HIS 122<br>ILE 124<br>ASP 125<br>LYS 546
Meclofenamic acid	-	-	-	-	-	-	-	-	-	-	-	-	-	-	-	-8.477	1	VAL 344
**VOMs**																		

3-Carene	-6.627	0	-	-5.619	0	-	-6.966	0	-	-6.714	0	-	-6.073	0	-	-6.356	0	-
β-Myrcene	-5.655	0	-	-4.643	0	-	-5.298	0	-	-5.931	0	-	-5.361	0	-	-5.326	0	-
Limonene	-6.501	0	-	-5.854	0	-	-6.853	0	-	-7.103	0	-	-6.607	0	-	-6.710	0	-
Eucalyptol	-5.211	0	-	-5.657	0	-	-6.520	3	SER 59<br>TYR 60<br>LYS 296	-5.263	0	-	-5.802	0	-	-6.174	0	-
γ-Terpinene	-6.505	0	-	-5.89	0	-	-6.845	0	-	-7.247	0	-	-6.283	0	-	-6.561	0	-
p-Cymene	-6.759	0	-	-5.931	0	-	-6.890	0	-	-7.331	0	-	-6.656	0	-	-6.555	0	-
β-Thujone	-6.148	0	-	-5.769	0	-	-6.387	3	SER 59<br>GLN 65<br>MET 436	-6.201	1	TRP 90	-5.582	0	-	-6.320	0	-
Isoborneol	-5.180	2	ASN 233	-5.669	1	TYR 332	-6.506	3	ILE 198<br>GLY 205<br>GLN 206	-5.483	2	THR 376<br>VAL 465	-5.508	0	-	-5.782	1	VAL 349
Geranial	-4.572	0	-	-4.727	0	-	-5.819	1	TYR 188	-6.431	1	TYR 491	-5.816	1	ARG 221	-5.625	1	TYR 348
Cuminaldehyde	-6.344	0	-	-6.131	1	TYR 440	-6.932	0	-	-7.220	0	-	-6.582	0	-	-6.516	0	-
β-Caryophyllene	-8.240	0	-	-7.083	0	-	-7.792	0	-	-6.981	0	-	-6.874	0	-	-7.439	0	-
γ-Curcumene	-6.988	0	-	-6.192	0	-	-8.208	0	-	-7.975	0	-	-7.099	0	-	-6.897	0	-
Germacrene D	-6.631	0	-	-6.721	0	-	-7.935	0	-	-6.561	0	-	-6.882	0	-	-6.658	0	-
τ-Cadinol	-7.552	3	SER 293<br>TYR 341	-7.310	1	THR 120	-7.516	2	TYR 60<br>LYS 296	-7.695	1	VAL 352	-6.736	0	-	-7.521	0	-
(*E*)-2-Decen-1-ol	-4.821	4	TYR 124<br>TYR 337<br>TYR 341	-4.289	3	PHE 371<br>HIS 372<br>ASP 375	-5.473	2	CYS 172<br>TYR 435	-4.464	2	SER 118<br>GLN 478	-4.689	2	ARG 221<br>GLN 656	-4.549	1	SER 353
Decanal	-4.600	0	-	-3.948	0	-	-4.698	1	CYS 172	-4.773	2	GLY 371<br>MET 374	-4.409	1	ILE 320	-4.943	0	-

(*E*)-2-Decenal	-4.923	1	PHE 295	-4.144	1	THR 120	-5.267	1	CYS 397	-4.641	1	THR 376	-5.112	0	-	-4.921	2	ALA 516<br>ILE 517
Myristicin	-6.155	3	TYR 124<br>PHE 295<br>ARG 296	-6.290	1	THR 120	-6.815	4	ARG 42<br>LYS 296<br>CYS 397	-7.203	0	-	-5.911	3	TYR 81<br>TYR 383	-6.782	1	PHE 529
Nonyl acetate	-5.071	3	TYR 124<br>PHE 295<br>ARG 296	-4.204	0	-	-4.906	1	CYS 397	-4.664	0	-	-4.111	1	THR 366	-5.546	1	PHE 529
(*Z*)-Methyl isoeugenol	-5.724	1	PHE 295	-5.579	3	THR 120<br>SER 198	-6.483	1	TYR 435	-6.351	1	CYS 200	-5.654	2	THR 366<br>ARG 457	-6.272	0	-
3-Allylguaiacol	-5.870	2	PHE 295<br>ARG 296	-5.721	2	SER 198<br>ALA 199	-6.718	1	VAL 235	-6.855	3	CYS 200<br>SER 242<br>ASN 370	-5.331	4	ARG 370<br>SER 447<br>THR 545	-6.068	0	-
2-Methyl-2,3-dihydrobenzofuran	-6.216	1	TYR 124	-6.018	0	-	-7.138	2	GLY 12<br>ALA 35	-7.223	1	SER 242	-5.972	0	-	-6.183	0	-
Dipropyl disulfide	-3.116	0	-	-3.210	0	-	-4.086	0	-	-3.880	0	-	-3.529	0	-	-3.614	0	-
Dipropyl trisulfide	-3.160	0	-	-3.351	0	-	-3.935	0	-	-4.125	0	-	-3.565	0	-	-3.826	0	-
